# A nested leucine rich repeat (LRR) domain: The precursor of LRRs is a ten or eleven residue motif

**DOI:** 10.1186/1471-2180-10-235

**Published:** 2010-09-09

**Authors:** Norio Matsushima, Hiroki Miyashita, Tomoko Mikami, Yoshio Kuroki

**Affiliations:** 1 Sapporo Medical University Center for Medical Education, Sapporo, Hokkaido 060-8556, Japan; 2 Sapporo City University School of Nursing, Sapporo, Hokkaido 060-0011, Japan; 3 Sapporo Medical University School of Medicine, Sapporo, Hokkaido 060-8556, Japan

## Abstract

**Background:**

Leucine rich repeats (LRRs) are present in over 60,000 proteins that have been identified in viruses, bacteria, archae, and eukaryotes. All known structures of repeated LRRs adopt an arc shape. Most LRRs are 20-30 residues long. All LRRs contain LxxLxLxxNxL, in which "L" is Leu, Ile, Val, or Phe and "N" is Asn, Thr, Ser, or Cys and "x" is any amino acid. Seven classes of LRRs have been identified. However, other LRR classes remains to be characterized. The evolution of LRRs is not well understood.

**Results:**

Here we describe a novel LRR domain, or nested repeat observed in 134 proteins from 54 bacterial species. This novel LRR domain has 21 residues with the consensus sequence of LxxLxLxxNxLxxLDLxx(N/L/Q/x)xx or LxxLxCxxNxLxxLDLxx(N/L/x)xx. This LRR domain is characterized by a nested periodicity; it consists of alternating 10- and 11- residues units of LxxLxLxxNx(x/-). We call it "IRREKO" LRR, since the Japanese word for "nested" is "IRREKO". The first unit of the "IRREKO" LRR domain is frequently occupied by an "SDS22-like" LRR with the consensus of LxxLxLxxNxLxxLxxLxxLxx or a "Bacterial" LRR with the consensus of LxxLxLxxNxLxxLPxLPxx. In some proteins an "SDS22-like" LRR intervenes between "IRREKO" LRRs.

**Conclusion:**

Proteins having "IRREKO" LRR domain are almost exclusively found in bacteria. It is suggested that IRREKO@LRR evolved from a common ancestor with "SDS22-like" and "Bacterial" classes and that the ancestor of IRREKO@LRR is 10 or 11 residues of LxxLxLxxNx(x/-). The "IRREKO" LRR is predicted to adopt an arc shape with smaller curvature in which β-strands are formed on both concave and convex surfaces.

## Background

LRR (leucine rich repeat) domains are present in over 60, 000 proteins listed in PFAM, PRINTS, SMART, InterPro and PANTHER databases [1]. LRR-containing proteins have been identified in viruses, bacteria, archae, and eukaryotes. Most LRR proteins are involved in protein, ligand and in protein, protein interactions; these include plant immune response and the mammalian innate immune response [2-6].

All LRR units can be divided into a HCS (highly conserved segment) and a VS (variable segment). The HCS part consists of an eleven residue stretch, LxxLxLxxNxL, or a twelve residue stretch, LxxLxLxxCxxL, in which "L" is Leu, Ile, Val, or Phe, "N" is Asn, Thr, Ser, or Cys, and "C" is Cys, Ser or Asn. Three residues at positions 3 to 5 in the highly conserved segments form a short β-strand. The β-strands stack parallel and the multiple LRRs then form an arc. The concave face consists of a parallel β-sheet and the convex face is made of a variety of secondary structures including the a-helix, 3_10_-helix, polyproline II helix, and an extended structure or a tandem arrangement of β-turns. In most LRR proteins the β-strands on the concave surface and (mostly) helical elements on the convex surface are connected by short loops or β-turns. Seven classes of LRRs have been recognized, characterized by different lengths and consensus sequences of the VS part of the repeats [7,8]. They are "RI-like", "CC", "Bacterial", "SDS22-like", "plant specific", "typical", and "TpLRR"[3]. The seven classes of LRR domains adopt a variety of structures.

"Typical" LRRs are the most abundant LRR class. The consensus sequence is **L**xx**L**x**L**xx**N**x**L**xx**L**pxxoFxx**L**xx. The repeat length is 20-27 residues. Bold uppercase letters indicate more than 70% occurrence of a given residue in a certain position; normal letters indicate 40-70% occurrence and lowercase letters indicate 30-40% occurrence; "o" indicates a non-polar residue, and "x" indicates nonconserved residues. Their variable segments adopt mainly polyproline II plus β-turn, consecutive β-turns or β-turn plus polyproline II in the convex faces; the structural units may be represented by β - (β_t _+ PPII). "RI-like" LRRs are contained in proteins such as ribonuclease inhibitor and Ran GTPase activating protein. The consensus sequence is **L**xx**L**x**L**xx**N**x(**L/C**)xxxgoxxLxxoLxxxxx. The repeat length is 28-29. Their VSs mainly adopt α-helix (β - α structural units). Cysteine-containing (CC) LRR proteins include GRR1 proteins from *Saccharomyces cerevisiae*. The consensus sequence is **L**xx**L**x**L**xx**C**xx**I**T**D**xxoxxL(a/g)xx(**C/L**)xx. The repeat length is 25-27. Their VSs mainly adopt α-helix (β - α structural units). A GALA-LRR is a subclass of CC-LRR; its consensus sequence is **L**xx**L**x**L**xx**N**x**Igd**x(g/a)axxLax(n/s/d)xx of 24 residues [9]. Plant-specific (PS) LRR proteins include PGIP and Cf-2.1. The consensus sequence is **L**xx**L**x**L**xx**N**x**L(**t/s)**G**x**IP**xxLGxLxx. The repeat length is 23-25. The VSs mainly adopt 3_10 _- helix. Also in individual LRRs the β-strand on the concave face at the N-terminus and the 3_10 _- helix on the convex face at the C-terminus is connected by a β-turn; the structural units are β - (β_t _+ 3_10_). "SDS22-like" LRRs are included in SDS22 and internalins. The consensus sequence is **L**xx**L**x**L**xx**N(**r/k)**I(**r/k)(r/k)IE(N/G)LEx**L**xx. The repeat length is 21-23. The structural units of individual repeats are β - 3_10_. "Bacterial" LRRs are found in YopM from *Yersinia pestis*, and IpaH from *Shigella flexneri*. The consensus sequence is **L**xx**L**x**V**xx**N**x**L**xx**LP(**D/E)**LP**xx. The repeat length is 20-22. The structural units are β - pII. "TpLRR" are found in *Treponema pallidum *LRR protein and in *Bacteroides forsythus *surface antigen. The consensus sequence is **L**xx**L**xLxxx**L**xxIgxxAFxx(C/N)xx. The repeat length is 23-25. The dominant feature is a highly conserved segment of ten residues, differing from the corresponding eleven residues of other LRRs. The structure of this class remains unknown.

Most of the known LRR structures have a cap, which shields the hydrophobic core of the first unit of LRR domain at the N-terminus and/or the last unit at the C-terminus. In extracellular proteins or extracellular regions, these caps frequently consist of Cys clusters including two or four Cys residues; the Cys clusters on the N- and C-terminal sides of the LRR arcs are called LRRNT and LRRCT, respectively [4-6]. Non-LRR, island regions interrupting LRRs are widely distributed. Island regions are observed in many LRR proteins including plant LRR-RLKs, plant LRR-RLPs, insect Toll and Toll-related proteins, Slit proteins, fungi adenylate cyclases, and *Leishmania *proteophosphoglycans [10-14].

The evolution of LRRs is not well understood. It is not even known whether all LRR's share a common ancestor. Kobe and Deisenhofer [2] pointed out the possibility of their having been at least a few independent occurrences of LRRs. Kajava [7] also suggested separate origins for several different classes of LRRs based on the high levels of conservation within each LRR class. In contrast, Andrade et al., [15] found that searches by a homology-based method, REP, could not absolutely partition LRRs into these separate classes and thus they suggested that these proteins have a common origin, rather than separate origins as proposed by Kajava.

Duplication and recombination as a mechanism of the evolution of the disease resistance gene (R-gene) from various plant species has been proposed by many investigators [16-24]. Distinct higher-order repeating units of LRR's occur in a group of LRR proteins including ribonuclease inhibitor, the subfamily of small leucine-rich repeat proteoglycan (SLRP), and the subfamily of Toll-like receptors (TLR7, TLR8 and TLR9) [4,25-28]. An evolutionary model has been proposed that involves duplication of the higher-order LRR repeating units [26,28]. Moreover, the possibility of horizontal gene transfer (HGT) has been discussed [29].

**Figure 1:**
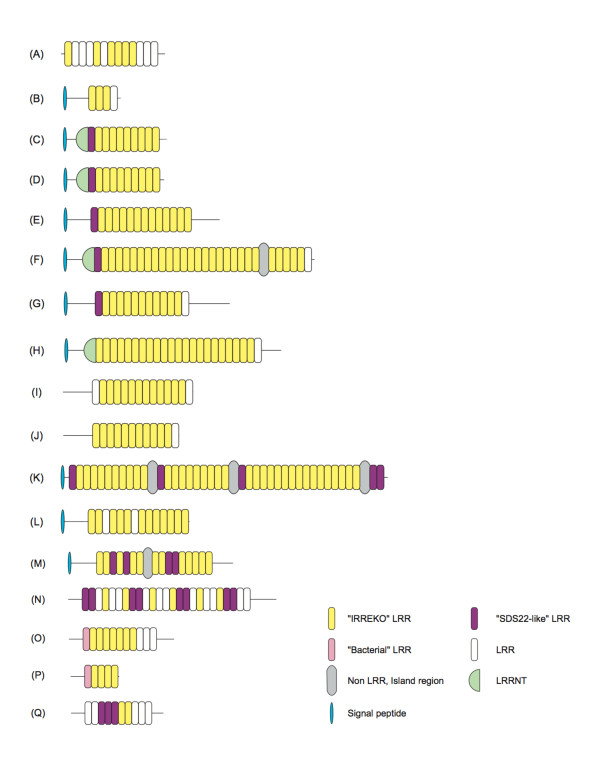
**Schematic representation of seventeen, representative proteins having IRREKO LRRs**. **(A)***Escherichia coli *yddk; **(B)***Bifidobacterium animalis *BIFLAC_05879; **(C)***Vibrio harveyi HY01 *A1Q_3393; **(D)***Shewanella woodyi ATCC 51908 *SwooDRAFT_0647; **(E)***Unidentified eubacterium SCB49 *SCB49_09905; **(F)***Colwellia psychrerythraea *CPS_3882; **(G)***Listeria monocytogenes *lmo0331 protein; **(H)***Treponema denticola *TDE_0593; **(I)***Polaromonas naphthalenivorans *Pnap_3264; **(J)***Ddelta proteobacterium MLMS-1 *MldDRAFT_4836; **(K)***Kordia algicida OT-1 *KAOT1_04155; **(L)***Coprococcus eutactus ATCC 27759 *COPEUT_03021; **(M)***Clostridiales bacterium 1_7_47_FAA *Cbac1_010100006401; **(N)***Listeria *lin1204/LMOf6854_0364; **(O)***Escherichia coli SMS-3-5 *EcSMS35_1703; **(P)***Escherichia coli O157:H7 *ECS2075/Z2240; **(Q)***Trichomonas vaginalis G3 *TVAG_084780. Symbol "□" indicates LRR that appears not to belong to the known seven classes and IRREKO motif.

*Escherichia coli *yddk is 318 residues long and contains 13 tandem repeats of LRRs; six of the 13 repeats have the consensus of LxxLxLxxNxLxxLxLxxxxx with 21 residues (Figure 1A). The variable segment differs significantly from those of the above seven classes. The purpose of this paper is to investigate the occurrence of this novel domains. We identified many LRR proteins having the novel domain (called IRREKO@LRR) and analyzed their sequences. We discuss the evolution and structure of "IRREKO" LRR.

## Results

### Proteins having IRREKO@LRRs

We identified a total of 134 IRREKO@LRR proteins from 54 bacterial species including *Escherichia, Shigella, Vibrio, Shewanella, Photobacterium, Bifidobacterium, Porphyromonas, Treponema, Listeria, Alistipes, Bacteroides, Clostridium, Cytophaga*, and *Flavobacterium* (Additional file 1, Table 1). A group of these proteins contain a signal peptide (but have no transmembrane helix), indicating that they are extracellular. The others lack both a signal peptide and a transmembrane helix, indicating that they are intracellular.

Some extracellular IRREKO@LRR proteins contain Cys clusters on the N-terminal side of the IRREKO@LRR domain (LRRNT); while LRRCT is not observed. For examples, IRREKO@LRR proteins from *Vibrio*, *Shewanella*, and *Photobacterium *have an LRRNT with the pattern of *Cx_16_C* (Additional file 1, Table 1). Three *Vibrio *IRREKO@LRR proteins (VV2_1682, CPS_3882 and VVA0501) have an LRRNT of *Cx_20_C*. Cysteine in the first LRR sometimes participates in LRRNT (Figure 1).

Some IRREKO@LRR proteins have non-LRR, island regions interrupting LRRs (Figure 1 and Additional files 1 and 2: Table 1 and Figure S1, respectively). They include KAOT1_04155 from *Kordia algicida OT-1*, CPS_3882 from *Vibrio psychroerythus*, Fjoh_1188/FjohDRAFT_4748 and Fjoh_1189/FjohDRAFT_4747 from *Flavobacterium johnsoniae*, Cbac1_010100006401 from *Clostridiale bacterium 1_7_47_FAA*, SCB49_05520 from *unidentified eubacterium*, ALIPUT_01468 from *Alistipes putredinis*, and FAEPRAM212_00794 from *Faecalibacterium prausnitzii M21/2*.

There is a single example of an "IRREKO" domain from a eukaryote and a single example from a virus. The eukaryote protein is TVAG_084780 from *Trichomonas vaginalis G3 *(Figure 1Q and Additional file 2, Figure S1). TVAG_084780 contains 10 LRRs. Two of the 10 repeats are clearly "IRREKO" domains. The virus protein is MSV251 from *Melanoplus sanguinipes entomopoxvirus *[Q9YVJ1]. This protein contains 11 LRRs with the consensus of **L**ky**L**d**C**s**NN**x**L**xn**L**xiN(n/d)n (Additional file 1, Table 1). The repeating unit length is 19 residues and thus shorter than that of typical "IRREKO" LRR.

### Two subtypes of IRREKO@LRR domains

IRREKO@LRRs that are 21 residues long may be classified into two subtypes (Figure 1). The first subtype has the consensus of LxxLxLxxNxLxxLDLxx(N/L/Q/x)xx, while the second has the consensus of LxxLxCxxNxLxxLDLxx(N/L/x)xx, where "L" is Leu, Val, Ile, Phe, Met or Ala, "N " is Asn, Thr or Ser, "D" is Asp or Asn, "Q" is Gln, and "x" is nonconserved residues. As well as the other seven classes, "x" is generally hydrophilic or neutral residues (Figure 1 and Additional files 1 and 2: Table 1 and Figure S1, respectively).

In these two subgroups, "L" at positions 1, 4, 14 and 16 is predominantly Leu, while "L" or "C" at position 6 is not only Leu or Cys but also Val or Ile, and frequently Ala and Phe. "N" at position 9 is predominantly Asn and often Thr, Ser or Cys. "D" at position 15 is predominantly occupied by Asp and frequently by Asn. Position 19 is often occupied by Leu, Asn, or Gln. Some IRREKO@LRR proteins such as *Listeria *internalin-J homologs and four *Bacteroides *proteins include LRRs in which the HCS part consists of a twelve residue stretch, LxxLxLxx(N/C)xxL As LRRs with 20 or 22 residues sometimes keep the most conserved segments of Lx(L/C) in both HCS and VS parts, we regard those as IRREKO@LRR.

IRREKO@LRR domains that mainly consist of the first subtype are observed in 61 proteins (Additional file 1, Table 1). Some proteins have the consensus of LxxLxLxxNxLxxLDLxxNxx. These include BIFLAC_05879 and BLA_0865 from *Bifidobacterium animalis*, A1Q_3393, VAS14_09189, VAS14_14509, and CPS_2313 from *Vibrio *species, SwooDRAFT_0647, SwooDRAFT_0647, and Shal_3481 from *Shewanella *species, and SKA34_06710 and SKA34_09358 from *Photobacterium sp. SKA34 *(Figures 1B, C and 1D, and Additional file 2, Figure S1). Also, the consensus of LxxLxLxxNxLxxLDLxxLxx is observed in a few proteins including SCB49_09905 from *unidentified eubacterium SCB49 *(Figure 1E). The pattern of LxxLxLxxNxLxxLDLxxQxx is observed in only CPS_3882 from *Vibrio psychroerythus *(Figure 1F).

IRREKO@LRR domains that consist mainly of the second subtype are observed in 57 proteins (Additional file 1, Table 1). The consensus of LxxLxCxxNxLxxLDLxxNxx in which "L" at position 16 is more frequently occupied by Val or Ile than by Leu is observed in some proteins. They include *Listeria *lmo0331 homologs, CHU_0515 from *Cytophaga hutchinsonii *and PORUE0001_1723 from *Porphyromonas uenonis 60-3 *(Figure 1G). Also, the pattern of LxxLxCxxNxLxxLDLxxLxx is observed in TDE_0593, TDE_2231, and TDE_2003 from T*reponema denticola *(Figure 1H, and Additional file 2, Figure S1). Moreover, the pattern of LxxLxCxxNxLxxLDLxxVxx is observed in Pnap_3264 from *Polaromonas naphthalenivorans *and MldDRAFT_4836 from *Delta proteobacterium MLMS-1 *(Figures 1I and 1J, and Additional file 2, Figure S1).

The coexistence of the first and the second subtypes is observed in the LRR domains in at least six IRREKO@LRR proteins. They include KAOT1_04155 from *Kordia algicida OT-1*, COPEUT_03021 from *Coprococcus eutactus ATCC 27759*, Fjoh_1188/FjohDRAFT_4748 and Fjoh_1189/FjohDRAFT_4747 from *Flavobacterium johnsoniae*, RUMGNA_03120 from *Ruminococcus gnavus ATCC 29149*, DORFOR_03338 from *Dorea formicigenerans ATCC 27755*, and internain-J homologs from eleven *Listeria monocytogenes *strains (Figures 1K and 1L, and Additional file 2, Figure S1).

### Nested periodicity of IRREKO@LRRs

IRREKO@LRRs show a characteristic, nested periodicity; the domains consist of alternating 10- and 11- residue units of LxxLxLxxNx(x/-). To confirm this periodic nesting we performed detailed sequence analysis of IRREKO@LRR proteins using dot plots analysis and a radar chart analysis.

Self dot plots were performed for four IRRECO@LRR proteins - BIFLAC_05879 from *Bifidobacterium animalis*, A1Q_3393 from *Vibrio harveyi HY01*, lmo0331 protein from *Listeria monocytogenes *and an internalin-related protein, TDE_0593, from *Treponema denticola *- (Additional file 3, Figure S2). The self dot plots indicate that these proteins demonstrate tandem repeats of short residues that is ~10-11 residues long, in addition to tandem repeats of IRRECO@LRR with 21 residues.

**Figure 2:**
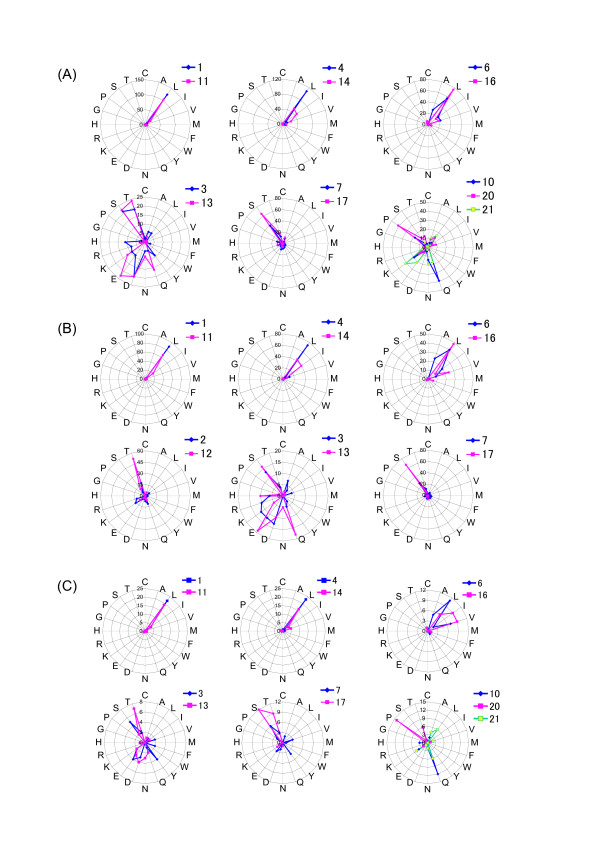
**Radar charts of IRREKO@LRRs in three families**. **(A)** Twelve proteins from seven *Vibrio *species; **(B)** Nine, potential homologs from four *Shewanella *species; **(C)** Four, potential homologs from two *Photobacterium *species. For radar charts, 137 IRREKO@LRRs in the *Vibrio *proteins, 82 repeats in the *Shewanella *proteins and 26 repeats in the *Photobacterium *proteins, which are all 21 residues long, were used. The length of each ray indicated by blue or pink is the occurrence frequency of each amino acid at two or three positions of "IRREKO" LRR whose a consensus sequence is L^1^x^2^x^3^L^4^x^5^L^6^x^7^x^8^N^9^x^10^L^11^x^12^x^13^L^14^x^15^L^16^x^17^x^18^x^19^x^20^x^21^.

Radar charts were drawn for three families of IRREKO@LRRs proteins, in which the occurrence frequency of amino acids is compared between positions 1-10 and positions 11-21. Figure 2A shows a radar chart of *Vibrio *proteins. Seven *Vibrio *species encode twelve IRREKO@LRR proteins which are potential homologs (Additional file 1, Table 1). The IRREKO@LRRs domains in their proteins contain 158 LRR repeats. One hundred thirty-seven of the 158 repeats are complete "IRREKO" domains with 21 residues. The radar chart of the 137 LRRs is shown in Figure 2. As expected, "L" at positions 1, 4, and 6 is highly conserved with positions 11, 14 and 16, respectively. In addition, a significant, weak conservation is observed between positions 10 and 21 but not 20, because amino acid distribution of positions 10 and 21 is very similar and are relatively rich in Lys, Asn and Gln. Alsor, positions 3 and 13 show a conservation in which the amino acids are relatively rich Ser, Thr, Asp and Glu. Moreover, positions 7 and 17 show a weak conservation at which those are relatively rich in Ser and Thr.

Similarly, in addition to high conservation of positions of 1-11, 4-14, and 6-16, a weak conservation among even "x" positions occupied by non-conserved residues is also observed in IRREKO@LRRs within nine, potential homologs from four *Shewanella *species; positions 2-12, 3-13, and 7-17 are relatively rich in Thr and Ser, and in those within four, potential homologs from two *Photobacterium *species; positions 3-13 are relatively rich in Thr, Ser, Asp and Glu, and positions 7-17 are relatively rich in Ser and Thr, and positions 10-21 are relatively rich in Gln and Lys (Figures 2B and 2C).

The analyses of both dot plots analysis and radar chart demonstrate that IRREKO@LRRs show a nested periodicity consisting of alternating 10- and 11- residue units with the consensus of LxxLxLxxNx(x/-).

### Secondary structure prediction

The protein secondary structure prediction of IRREKO@LRR proteins was performed (Additional file 4, Figure S3). *E. coli *yddk contains 13 LRRs (Figure 1A). Proteus and SSpro4.0 [30,31] predict that 12 of the 13 LRRs prefer β-strands at positions 3 through 5 and/or its neighboring positions in the HCS part; although only the eighth LRR does not prefer β-strand, its HCS part - **V**TY**F**S**A**AH**N**Q**L**- is clearly a canonical LRR. Similarly, all or most LRRs in other proteins prefer β-strands at the corresponding positions in the HCS part.

Both methods of secondary structure prediction indicate that residues at positions 13 through 15 and/or its neighboring positions prefer coil conformations in most LRRs of *E. coli *yddk, *Listeria *lmo0331 protein, and *Treponema *TDE_0593. On the other hand, in most LRRs of *Bifidobacterium *BIFLAC_05879, *Vibrio *A1Q_3393 and *Shewanella *SwooDRAFT_0647, residues at the corresponding positions prefer β-strands. It is concluded that individual three residues at positions 3 to 5 and 13 to 15 could form a short β-strand.

### Occurrence of "SDS22-like" and "Bacterial" LRR domains within IRREKO@LRR domains

The first LRR of LRR domain in a large number of IRREKO@LRR proteins are an "SDS22-like" domain, LxxLxLxxNxLxxLxxLxxLxx; even though "N" at position 9 is sometimes occupied by Lys, Gln or Leu (which is frequently seen in the first LRR of LRR domains consisting of only other LRR classes) (Additional file 1, Table 1)[27]. Their proteins include eleven proteins from seven *Vibrio *species, eight proteins from five *Shewanella *species, eleven internalin-J homologs from eleven *Listeria monocytogenes strains*, nine lmo0331 homologs from eight *L. monocytogenes *strains and *L. innocua*, and nine proteins from three *Flavobacterium *species.

"SDS22-like" LRR occurs even in the middle position in the IRREKO@LRR domains in some proteins. Cbac1_010100006401 from *Clostridiale bacterium 1_7_47_FAA *with 1,002 residues contains 16 tandem repeats of LRRs; one non-LRR, island region is observed between the seventh and eighth LRRs (Figure 1M, and Additional file 2, Figure S1). Twelve of the 16 repeats are "IRREKO" domain with 20-22 residues. On the other hand, the remaining (LRRs 3, 5, 10 and 11) belong to "SDS22-like" class with the consensus is LxxLxCxxNxLxxLxxLxxLxx.

The three *Listeria *lin1204 homologs - LMOf6854_0364, LMOh7858_0369, and LMOf2365_0349 - have 993-1,099 residues and contain 25 tandem repeats of LRRs (Figure 1N and Additional file 2, Figure S1). Six of the 25 repeats are "IRREKO" domain, while eight repeats are "SDS22-like" class.

Other examples include FB2170_11006 from *Flavobacteriale bacterium HTCC2170 *and three proteins - BACOVA_03150 from *Bacteroides ovatus*, BACCAC_03004 from *Bacteroides caccae ATCC 43185*, and BACFIN_03505 from *Bacteroides finegoldii DSM 17565 *- that are homologous to each other (Additional file 1, Table 1). The former contains nine tandem repeats of LRRs and the third LRR of **L**VL**V**E**I**LA**N**E**L**HT**I**KG**L**SK**M**TQ is an "SDS22-like" class. The latter three proteins contains eight tandem repeats of LRRs. The fifth LRR is **I**A**I**L**I**G**C**A**F**QS**L**D**I**L**C**CPS and thus appears to be a "SDS22-like" domain.

Five ECUMM_1703 homologs from three *Escherichia coli *strains and two *Shigella *species contain 11-15 tandem repeats of LRRs (Figure 1O and Additional file 1, Table 1). Three ECs2075/Z2240 homologs from several *Escherichia coli *strains and two *Shigella *strains contain four or five tandem repeats of LRRs (Figure 1P and Additional file 1, Table 1). The first LRR are all **M**AS**L**D**L**SY**L**D**L**SE**LP**P**IP**ST and thus belongs to "Bacterial" class with the consensus of LxxLxLxxNxLxxLPxLPxx (although "N" at position 9 is often occupied by Leu) [27]. Three ECUMM_1723 homologs occur in three *E. coli *strains with 11 repeats of IRREKO@LRR. The first LRR is QND**I**D**L**SG**L**N**L **(T/S)TQ**P**PG**L**QN. It may belong to "Bacterial" LRR.

## Discussion

### IRREKO@LRR as new class of LRR

The present observations indicate that IRREKO@LRR is a new class of LRR. This is supported by several additional observations. The identification of LRRs by PFAM or SMART occurs in a large number of IRREKO@LRR proteins including *E. coli *yddK; this results from the significant similarity of their HCSs with those of the other LRR classes. There are many LRR proteins that contain the LRR domain consisting mainly of "SDS22-like" domain. The "SDS22-like" LRRs in *Listeria *lin1204/LMOf6854_0364 and *Microcoleus chthonoplastes PCC 7420 *MC7420_1958 [B4VM60] also have some IRREKO@LRR domains.

### Evolution

**Figure 3:**
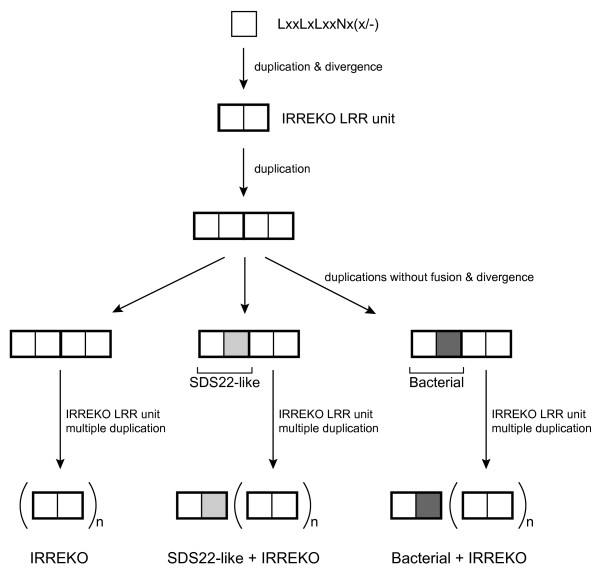
**Evolution of LRR proteins containing "IRREKO", "SDS22-like" and "Bacterial" LRR classes**. Light gray squares indicate the variable segment of "SDS22-like" LRR class and dark gray squares indicate the variable segment of "Bacterial" LRR class. "n" indicate the repeat number of "IRREKO" LRRs

The IRREKO@LRRs show a nested periodicity consisting of alternating 10- and 11- residue units with the consensus of Lxx(L/C)xLxxNx(x/-). The IRREKO@LRR domains in many proteins contain a mixture of both subtypes. The first LRR of the LRR domains is frequently "SDS22-like" or "Bacterial" classes. In addition, among the IRREKO@LRR domain "SDS22-like" class occurs in some proteins. The two subtypes of IRREKO@LRR appear to have evolved from a common precursor. Further, the "IRREKO" domain evolved from a precursor common to "SDS22-like" and "Bacterial" classes. The precursor of IRREKO@LRR is shorter sequence - LxxLxLxxNx(x/-) -. This parsimonious evolutionary scenario for three LRR classes, "IRREKO", "SDS22-like", and "Bacterial" LRRs is shown in Figure 3.

Previous studies revealed that LRR domains in many LRR proteins contain tandem repeats of a super-domain of ***STT***, where "***T***" is "typical" LRR and "***S***" is "Bacterial" LRR; they include the SLRP subfamily (biglycan, decorin, asporin, lumican, fibromodulin, PRELP, keratocan, osteoadherin, epiphycan, osteoglycin, opticin, and podocan), the TLR7 family (TLR7, TLR8 and TLR9), the FLRT family (FLRT1, FLRT2, and FLRT3), and OMGP [4,25-27]. The combination of the previous and the present observations suggest that the four LRR classes of "Bacterial", "typical", "SDS22-like" and "IRREKO" might evolve from a common precursor.

### Structure

**Figure 4:**
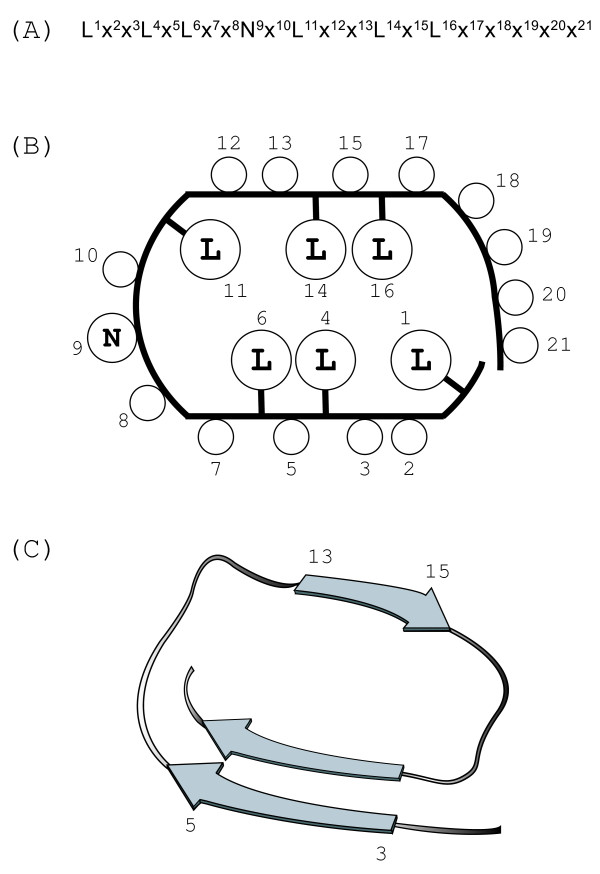
**Possible structure of IRREKO@LRRs**. **(A) **A consensus sequence of IRREKO@LRRs. Position 6 is occupied by not only Leu but also Cys. Position 19 is occupied by Asn, Leu, or Gln in some LRR domains. **(B) **2 D plot of the predicted side-chain orientation within one coil of the LRR superhelix. Location of the circles inside the coil contour indicates the occurrence in the interior of the structure. **(C) **Possible secondary structure of IRREKO@LRRs. Arrows represent β-strands.

The known LRR structures reveal that conserved hydrophobic residues in the consensus contribute to the hydrophobic cores in the LRR arcs [2-6]. As noted, the consensus of IRREKO@LRR is LxxLxLxxNxLxxLDLxx(N/L/Q/x)xx or LxxLxCxxNxLxxLDLxx(N/L/x)xx. It is likely that the conserved hydrophobic residues at the six (or seven) positions of 1, 4, 6 and 11, 14 and 16 (and 19) participate in the hydrophobic core (Figure 4).

The LRR structures with α-helices in their convex faces have more pronounced curvature than structures with 3_10 _or polyproline II helices [4,32]. This difference in curvature is attributed to the differences in diameter of the different secondary structure elements on the convex face, α-helices being wider than 3_10_-helices, polyproline II helices or tandem β-turns. IRREKO@LRR is predicted to adopt β-β structural units, because individual three residues at positions 3 to 5 and 13 to 15 could form a short β-strand (Figure 4). β-strands have the smallest diameter. Moreover, the loops that link the C-terminal ends of the β-strands in the HCS to the N termini of those in the VS appear to be different from the loops that link the C-terminal ends of those in the VS to the N termini of the following β-strands, as the HCS is one residue longer than the VS. Thus, an inferred arc structure of IRREKO@LRR has a smaller curvature.

Position 2 in the *i*-th and the (*i+1*)-th repeats of IRREKO@LRRs is alternatively occupied by positive and negative charged amino acids in some proteins. Examples include CdifQCD-2_010100017965 and CdifQ_04001775 from *Clostridium difficile *and CHU_1860 from *Cytophaga hutchinsonii*, as well as FjohDRAFT_1094 and Fjoh_0631 from *Flavobacterium johnsoniae* (Additional file 1, Table 1). The inferred arc structure of IRREKO@LRRs will enable them to form polar hydrogen bond interactions which lead to its structural stability.

It is possible that the β-solenoid structure of IRREKO@LRRs is related to β-helix proteins [33-35]. A β-β structural unit that is responsible for tandem repeats of GGxGxD is also observed in serralysin [36]. The β-solenoids with β-β structural units in IRREKO@LRR protein and serralysin represent an example of convergent evolution. Future studies should resolve this question.

## Conclusion

IRREKO@LRR is a new, unique class of LRR. IRREKO@LRR with the consensus of LxxLx(L/C) xxNxLxxLxLxx(L/Q/x)xx is a nested sequence consisting of alternating 10 - and 11-residue units of LxxLxLxxNx(x/-). The IRREKO@LRR domains frequently coexist with "SDS22-like" or "Bacterial" LRR. These findings suggest that the ancestor of IRREKO@LRR is shorter residues of LxxLxLxxNx(x/-) and that IRREKO@LRR evolved from a common ancestor with "SDS22-like" and "Bacterial" classes. IRREKO@LRRs are predicted to adopt an arc shape with smaller curvature in which individual repeats adopt β-β structural units.

## Methods

### IRREKO@LRR search

The putative uncharacterized protein yddK from *Escherichia coli *(*strain K12*) with 318 residues [YDDK_ECOLI] is an LRR protein. It is identified in the data bases of InterPro, PFAM, PRINTS and SMART. The InterPro data base indicates that the LRR domain contains nine repeats. The PFAM program predicts that yddK contain one significant LRR (residues 216-238) and seven insignificant LRRs (12-30; 33-53; 109-131; 153-175; 196-213; 260-282; 284-306).

We recently developed a new method that utilizes known LRR structures to recognize and align new LRR domains and incorporate multiple sequence alignments and secondary structure predictions [27]. This method predicts correctly the number of LRRs, their lengths and their boundaries. Its usefulness was confirmed by crystal structures of TLR1, TLR2, and TLR4 [37,38].

This new method was used for multiple sequence alignments of LRRs in the yddK protein. This analysis predicted not nine repeats of the LRRs but 13 repeats and also revealed that their "phasing" differ significantly. We noticed that LRRs, 1, 5 7, 8, 9, and 10 contain a unique domain whose consensus is LxxLxLxxNxLxxLxLxxxxx with 21 residues. The variable segment offers a characteristic hydrophobic pattern unidentified previously (Figure 1A). Each LRR domain is a nested sequence and consists of repeats alternating 10- and 11- residue units of LxxLxLxxNx(x/-).

LRR proteins having the IRREKO@LRR domains were identified in three steps:

**Step 1**: Detection of LRR proteins containing the six, novel LRRs in *E-coli *yddk by using FASTA

**Step 2**: Identification of the IRREKO@LRRs in individual LRR proteins by a new

method.

**Step 3: **Iteration of these two steps using novel LRRs in newly identified LRR proteins

In **step 1**, we performed similarity search using the six, novel LRRs as probes by FASTA at the Bioinformatic Center, Institute for Chemical Research, Kyoto University on April 27, 2009 http://www.genome.ad.jp/. This procedure detected many yddK homologs from *Escherichia coli strains *and *Shigella flexneri *[Q0T447 and Q83R94] with significant similarity (E-values < 6.5 × 10^-29^). In addition, two other proteins were detected with significant similarity (E-value < 3.3 × 10^-9^). One is SSON_1653 that is 387 residues long [Q3Z1L5]. The other is SD1012_2081 with 163 residues [B3WXZ7]. In **step 2**, we performed multiple sequence alignment among their LRR domains of SSON_1653 and Sd1012_2081. SSON_1653 contains 14 LRRs and 9 of the 12 repeats consist of LxxLxLxxNxLxxL(D/N)(L/F)xxxxx where "L" is Leu, Val, or Ile. Sd1012_2081 contains 4.5 LRRs; 3.5 of these repeats consist of LxxLxLxxNxLxxIx(I/A/F)xxaxx In **step 3**, the above procedures were iterated to identify other LRR proteins having this IRREKO@LRR domain.

### Sequence Analyses

The dot-matrix comparisons were performed using the BLOSUM62 scoring matrix and a window size of 21 residues http://emboss.bioinformatics.nl/cgi-bin/emboss/dotmatcher. A radar chart is a graphical method displaying multivariate data in the form of a two-dimensional chart of three or more quantitative variables represented on axes starting from the same point http://en.wikipedia.org/wiki/Radar_chart. For a given observation, the length of each ray is the occurrence frequency of each amino acid at two positions of "IRREKO" LRR with 21 residues. Multiple sequence alignments were performed by CLUSTALW at the Bioinformatic Center. The protein secondary structure prediction was performed by SSpro4.0 http://contact.ics.uci.edu/sspro4.html[30] and Proteus http://129.128.185.184/proteus/#[31]. Signal sequence analysis was carried out using the program SignalP [39].

## Authors' contributions

NM (corresponding author) carried out the molecular genetic studies, participated in the sequence alignment and drafted the manuscript. HM performed dot plot analysis and radar chart analysis. TM contributed to the data analysis including the sequence alignment. KY conceived of the study, and participated in its design and coordination. All authors read and approved the final manuscript.

## Supplementary Material

Additional file 1 **Table 1: IRREKO@LRR proteins**. **Database**; Protein accession number or identification number in EMBL or NCBI. **Consensus**; The consensus sequences of complete IRREKO@LRRs with 21 residues are shown. Bold uppercase letters indicate more than 60%, normal uppercase letters indicate more than 50% and less than 60%, and normal lowercase letters indicate less than more than 30% and less than 50%. "L" in the consensus sequence denotes Leu, Val, or Ile. "x" denotes any residues. **Length**; The length of complete amino acid sequences of proteins. **LRR repeat**; The repeat number of LRR domain. Number is the repeat number of complete IRREKO@LRRs with 21 residues. The numeral in the parenthesis is total repeat number of LRRs. **1st LRR**; The LRR class of the first repeat of LRR domain. **SIGNAL**; The Occurrence (○) and no-occurrence (-) of signal peptide sequence. **LRRNT**; The pattern of cysteine clusters of the N-terminal side of LRR domain. Click here for file

Additional file 2 **Figure S1: Sequence alignments of the LRR domain in seventeen IRREKO@ LRR proteins**. (A) *Escherichia coli *yddk; (B) *Bifidobacterium animalis *BIFLAC_05879; (C) *Vibrio harveyi *HY01 A1Q_3393; (D) *Shewanella woodyi *ATCC 51908 SwooDRAFT_0647; (E) *Unidentified eubacterium *SCB49 SCB49_09905; (F) *Colwellia psychrerythraea C*PS_3882; (G) *Listeria monocytogenes *lmo0331 protein; (H) *Treponema denticola *TDE_0593; (I) *Polaromonas naphthalenivorans *Pnap_3264; (J) *Ddelta proteobacterium *MLMS-1 MldDRAFT_4836; (K) *Kordia algicida *OT-1 KAOT1_04155; (L) *Coprococcus eutactus *ATCC 27759 COPEUT_03021; (M) *Clostridiales bacterium *1_7_47_FAA Cbac1_010100006401; (N) *Listeria *lin1204/LMOf6854_0364; (O) *Escherichia coli *SMS-3-5 EcSMS35_1703; (P) *Escherichia col*i O157:H7 ECS2075/Z2240; (Q) *Trichomonas vaginalis *G3 TVAG_084780. Overall consensus sequences of IRREKO@LRRs - LxxLxLxxNxLxxLDLxx(N/L/Q/x)xx or LxxLxLxxNxLxxLDLxx(N/L/Q/x)xx - are shown. The consensus amino acids are highlighted with reverse-contrast. Also the consensus amino acids of "SDS22-like" LRR with the consensus of LxxLxLxxNxLxxLxxLxxLxx and of "Bacterial" LRR with the consensus of LxxLxxNxLxxLPxLPxx are highlighted with reverse-contrast. Cysteines of the cysteine clusters at the N-terminal side of LRR domain are shown by underlined bold letter. **Cons**., the overall consensus sequences of IRREKO@LRRs; **SIGNAL**, signal peptide sequence; **LRR**; leucine rich repeat (LRR); **IRREKO**, IRREKO LRR; **SDS22**; "SDS22-like" LRR; **BAC**; "Bacterial" LRR; **ISLAND**, Island region interrupting LRRs; **N-TERM**, the N-terminal region of proteins; **C-TERM**, the C-terminal region of proteins; **LRRNT**; the region of cysteine clusters at the N-terminal side of LRR domain. Click here for file

Additional file 3 **Figure S2: Self-dot matrices for four IRREKO@LRR proteins**. (**A**) *Bifidobacterium animalis *BIFLAC_05879; (**B) ***Vibrio harveyi HY01*A1Q_3393, (**C**) *Listeria monocytogenes *lmo0331 protein; (**D) ***Treponema denticola *TDE_0593. A window size of 21 residues was used. The threshold is 30 in the upper panel and 10 or 15 in the lower panel. Residues used are full lengths for the self-dot matrices; residue 1-186, 1-278, 1-633, and 1-631 of BIFLAC_05879, *HY01*A1Q_3393, lmo0331 protein, TDE_0593, respectively, were used. The abscissa and the ordinate are residues number. Click here for file

Additional file 4 **Figure S3: Protein secondary structure prediction in five IRREKO@LRR proteins by the Proteus and SSpro4.0 programs**. **(A) ***Escherichia coli *yddk; **(B) ***Bifidobacterium animalis *BIFLAC_05879; **(C) ***Vibrio harveyi HY01 *A1Q_3393; **(D) ***Listeria monocytogenes *lmo0331 protein; **(E) ***Shewanella woodyi *ATCC 51908 SwooDRAFT_0647; **(F) ***Treponema denticola *TDE_0593. The highly conserved segment of individual LRRs is highlighted by a shadow. For comparison, its consensus sequence is shown in bold letters. Abbreviations: h/H, helix; c/C, coil; e/E, β-strand. Click here for file
