# Supplementary material for: A nested leucine rich repeat (LRR) domain: The precursor of LRRs is a ten or eleven residue motif
Source: BMC Microbiol. 2010 Sep 9;10:235. doi: 10.1186/1471-2180-10-235 (PMC2946307; doi:10.1186/1471-2180-10-235)
Supplement: Additional file 2 — Figure S1: Sequence alignments of the LRR domain in seventeen IRREKO@ LRR proteins. (A) Escherichia coli yddk; (B) Bifidobacterium animalis BIFLAC_05879; (C) Vibrio harveyi HY01 A1Q_3393; (D) Shewanella woodyi ATCC 51908 SwooDRAFT_0647; (E) Unidentified eubacterium SCB49 SCB49_09905; (F) Colwellia psychrerythraea CPS_3882; (G) Listeria monocytogenes lmo0331 protein; (H) Treponema denticola TDE_0593; (I) Polaromonas naphthalenivorans Pnap_3264; (J) Ddelta proteobacterium MLMS-1 MldDRAFT_4836; (K) Kordia algicida OT-1 KAOT1_04155; (L) Coprococcus eutactus ATCC 27759 COPEUT_03021; (M) Clostridiales bacterium 1_7_47_FAA Cbac1_010100006401; (N) Listeria lin1204/LMOf6854_0364; (O) Escherichia coli SMS-3-5 EcSMS35_1703; (P) Escherichia coli O157:H7 ECS2075/Z2240; (Q) Trichomonas vaginalis G3 TVAG_084780. Overall consensus sequences of IRREKO@LRRs - LxxLxLxxNxLxxLDLxx(N/L/Q/x)xx or LxxLxLxxNxLxxLDLxx(N/L/Q/x)xx - are shown. The consensus amino acids are highlighted with reverse-contrast. Also the consensus amino acids of "SDS22-like" LRR with the consensus of LxxLxLxxNxLxxLxxLxxLxx and of "Bacterial" LRR with the consensus of LxxLxxNxLxxLPxLPxx are highlighted with reverse-contrast. Cysteines of the cysteine clusters at the N-terminal side of LRR domain are shown by underlined bold letter. Cons., the overall consensus sequences of IRREKO@LRRs; SIGNAL, signal peptide sequence; LRR; leucine rich repeat (LRR); IRREKO, IRREKO LRR; SDS22; "SDS22-like" LRR; BAC; "Bacterial" LRR; ISLAND, Island region interrupting LRRs; N-TERM, the N-terminal region of proteins; C-TERM, the C-terminal region of proteins; LRRNT; the region of cysteine clusters at the N-terminal side of LRR domain. [file 1471-2180-10-235-S2.DOC]

(A)　 *Escherichia coli* yddk

**N-TERM** MITDLILHNHPR

## Cons. LxxLxLxxNxLxxLDLxxLxx

**N**

**1 IRREKO M**KT**I**T**L**ND**N**H**I**AH**LNA**KN**T**TK

**2 LRR L**EY**L**N**L**SN**N**N**L**LPTNDIDQLISS

**3 LRR** KHLWH**V**LV**N**G**I**NNDPLAQMQYWTAVRNIIDDTN

**4 LRR** EVT**I**D**L**SGLN**L**TTQPPGLQN

**5 IRREKO F**TS**I**N**L**DN**N**QFTH**FDA**TNYDR

**6 LRR L**VK**L**S**L**NS**N**A**L**ESINFPQGRNVS

**7 IRREKO I**TH**I**S**M**NN**N**A**L**RN**IDI**DR**L**SS

**8 IRREKO V**TY**F**S**A**AH**N**Q**L**EF**V**Q**L**ESCEW

**9 IRREKO L**QY**L**N**L**SH**N**Q**L**TD**I**V**A**GNKNE

**10 IRREKO L**LL**L**D**L**SH**N**K**L**TS**L**HNDL**F**PN

**11 LRR L**NT**L**L**I**NN**N**L**L**SEIKIFYSNFCN

**12 LRR V**QT**L**N**A**AN**N**Q**L**KYINLDFLTYLPS

**13**  **LRR I**KS**L**R**L**DN**N**K**I**THIDTNNTSD

**C-TERM**  IGTLFPIIKQSKT1

(B) *Bifidobacterium animalis* BIFLAC_05879

**SIGNAL** NTLKKMAVSAVAALAGVMMVAPTAMA

**N-TERM** ANGTIPIDAAHFPDTAFRVRIAQEFDSYPKDGKLTIAERNAVTEI

RGGDSYEIKFAKGIGYFPT

## Cons. LxxLxLxxNxLxxLDLxxNxx

**1 IRREKO L**EN**L**Y**L**YNQL**L**TS**IDL**SH**N**AK

**2 IRREKO L**QN**L**N**L**SR**N**K**L**TK**IDL**SR**N**PE

**3 IRREKO L**RD**L**D**L**SS**N**K**L**IS**INV**SK**N**TK

**4 LRR L**TE**F**D**F**QS**N**P**L**LWISSIAPAV

**C-TERM** NVDIYSPFTD

(C) *Vibrio harveyi HY01* A1Q_3393

**SIGNAL** MSRNKQKLVMATCGFLVSLNA

**LRRNT** FSGEVLDVSDIKFEDDNFQK**C**VLAQKVTNPTE

## Cons. LxxLxLxxNxLxxLDLxxNxx

**1 SDS22 I**TK**L**V**C**RQFN**I**DS**A**NE**I**HY**F**PA

**2 IRREKO L**KE**L**W**L**SGKQ**L**SN**IDL**Sk**N**LE

**3 IRREKO L**TS**I**S**I**TK**S**N**L**TS**LDL**SN**N**PN

**4 IRREKO L**TE**L**N**V**SF**N**Q**I**SE**LDL**TK**S**PK

**5 IRREKO I**TD**L**S**V**VS**N**Q**I**EA**LNL**SS**N**IE

**6 IRREKO L**KY**L**W**A**KE**N**K**L**KD**IDF**SH**N**PN

**7 IRREKO L**VQ**I**S**L**DN**N**Q**L**SK**VNI**SN**N**EQ

**8 IRREKO L**NK**L**S**L**MF**N**L**L**SS**LDV**SK**N**VK

**9 IRREKO L**TK**L**Y**V**TD**N**K**L**TK**LDV**SH**N**PK

**10 IRREKO L**RA**L**W**A**TE**N**P**L**ED**LDI**TS**N**SS

**C-TERM** LSDYEVDEGVLVQE

(D) *Shewanella woodyi ATCC 51908*　 SwooDRAFT_0647

**SIGNAL** MDYKQPLAMTLLSLAVSSSCF

**LRRNT** AADYQNVKDIPFKDSNFKA**C**VLAQTTEDPAA

## Cons. LxxLxLxxNxLxxLDLxx Nxx

**1 SDS22 I**TK**L**I**C**RQMK**I**NQ**V**DE**L**SH**F**PA

**2 IRREKO L**ER**L**W**I**SGPQ**L**KE**VDL**GSG**N**LK

**3 IRREKO L**TE**L**S**I**TKGK**L**TQ**LDL**SK **N**SQ

**4 IRREKO L**QE**L**N**I**SF**N**Q**I**SD**LDL**TH **N**PK

**5 IRREKO L**TD**I**S**V**VA**N**R**L**TE**LDL**SQ **N**SA

**6 IRREKO L**KY**L**W**A**KE**N**Q**F**TS**LDF**SH **N**SA

**7 IRREKO L**VQ**V**G**L**TS**N**Q**I**ST**IDV**SK **N**SE

**8 IRREKO L**NR**L**G**L**MY**N**Q**L**TK**LDV**SY **N**SK

**9 IRREKO L**TK**L**Y**L**TD**N**K**L**TH**IDI**SQ **N**PK

**10 IRREKO L**RD**L**W**A**TG**N**P**L**EQ**LDI**SK **N**PK

**C-TERM** LDDYEVDEGIKISE

(E) *Unidentified eubacterium SCB49* SCB49_09905

**SIGNAL** MKKIYLLIGIVFIGAQTMA

**N-TERM** QNIIFTDVNFKNTLLSSGPNSPVAWDNANNRIAIDVNNDGEIQQSEAML

## Cons. LxxLxLxxNxLxxLDLxxLxx

## N

**1 SDS22 V**YR**L**Q**V**DNEG**I**QD**V**NG**I**EF**F**TD

**2 IRREKO V**RI**L**N**V**NN**N**E**L**VD**LDV**TA**M**VN

**3 IRREKO L**EV**L**Y**M**GG**N**N**L**INP**NF**TN**L**PN

**4 IRREKO L**NL**V**Y**A**LD**N**P**I**TT**LDF**TG**V**SS

**5 IRREKO L**ET**L**L**L**GD**N**S**F**NA**LDI**SL**L**TN

**6 IRREKO L**KE**L**N**I**FGGA**I**TS**LDL**SN**Q**SL

**7 IRREKO L**EK**L**V**V**VN**T**Q**L**AS**INV**SA**L**QN

**8 IRREKO L**VE**L**S**L**IN**N**E**L**TT**LDV**SG**L**TS

**9 IRREKO L**EV**L**N**A**GG**N**N**I**NT**L**T**L**FN**N**PA

**10 IRREKO L**IG**L**N**A**FG**N**Q**L**SS**IDL**SQ**A**PA

**11 IRREKO L**TG**L**N**L**SD**N**L**F**TV**IDI**TV**L**SN

**12 IRREKO L**ID**L**A**V**HD**N**Q**L**SV**LDV**ST**N**ND

**13 IRREKO I**SE**L**S**A**TN**N**N**L**HF**LDL**SS**L**TG

**14 IRREKO L**IL**M**E**V**NN**N**N**L**TY**LNI**QN**T**VL

**C-TERM** SQDVDNHFFDENPDLLQICVDEGEIAFITQRALSYGYVNAVVEKC

VLGISETFANEIKLYPNPTVDTIYIESFSNLLRIEIYTLDGKKVA

NFKEIETSINISSLSSGTYILKLSTERGDVFEKIIKE

(F) *Vibrio psychroerythus* CPS_3882

**SIGNAL** MSIHFSIFKTLLFSFLVITLTAC

**LRRNT**  GGSGEESPEANQVEVIPPVTLPSVNEVPVITLNDEYSVIEYSDIT

VSALAEDSDGEIISYLWQQKSGLIVDLSETDSSTLTFTAPNVSED

QQLTFELTVTDDDNALTTKNIIIRLHALESDIEDIVFANENFKS**C**

IFNIAKEANLHKSVN

## Cons. LxxLxLxxNx LxxLDLxxQxx

## L

**1 SDS22 F**RQ**I**D**C**RDQN **I**SSTQG**L**EN**F**TE

**2 IRREKO L**KT**L**T**L**IN**T**Q **I**KT**IDL**SA**L**IR

**3 IRREKO L**ET**I**W**L**SR**N**Q **L**SE**IDL**SK**Q**TL

**4 IRREKO L**TD**L**E**L**HD**N**N **L**AS**INL**VN**Q**VL

**5 IRREKO L**TY**L**S**I**TN**N**Q **I**IS**IDL**AA**Q**TQ

**6 IRREKO L**TN**L**A**L**DG**N**Q **L**PE**VNL**SA**Q**KL

**7 IRREKO L**TH**L**Y**L**SN**N**Q **L**TK**VDL**SK**Q**KL

**8 IRREKO L**TD**L**A**L**SD**N**Q **L**TK**VDL**SE**Q**KL

**9 IRREKO L**TN**L**A**L**SN**N**Q **L**TE**VDL**STKPL

**10 IRREKO L**TH**L**R**L**ND**N**P **I**TD**IDL**RA**Q**RL

**11 IRREKO L**IL**L**G**L**SN**N**L **L**TE**VDL**RV**Q**EL

**12 IRREKO L**ID**L**D**L**SN**N**L **L**TE**VNL**RV**Q**EL

**13 IRREKO L**ID**L**D**L**SN**N**L **L**TG**VDL**RV**Q**EL

**14 IRREKO L**IG**L**D**L**SN**N**R **L**TE**VDL**ST**Q**TF

**15 IRREKO L**TG**L**N**L**DD**N**Q **L**TK**IDL**KN**Q**TK

**16 IRREKO L**QS**F**S**I**DN**N**Q **I**TE**LDL**SS**Q**PE

**17 IRREKO L**SR**I**S**I**WN**N**Y **L**TA**INL**STPLK

**18 IRREKO I**TD**L**D**L**TE**S**K **L**TT**IDL**TA**Q**PQ

**19 IRREKO L**KN**L**I**L**WN**N**E **L**TS**IDL**SN**L**VQ

**20 IRREKO L**ES**L**N**L**GS**N**DN**L**SE**VNL**AG**L**TG

**21 IRREKO L**SN**L**R**L**SNLN **L**ST**IDL**SQ**Q**SN

**22 IRREKO L**LS**L**H**I**DG**N**P **L**TT**LDL**SA**Q**KK

**23 IRREKO L**HT**L**S**I**SKGN **L**EN**FNL**TN**L**TK

**ISLAND**  LTSFASYNIEPETIQLFPDLK

**24 IRREKO L**DY**L**A**F**NYFQ AKY**INL**TAHTE

**25 IRREKO L**TT**L**V**I**NEGG **I**TD**IDL**SAHEK

**26 IRREKO I**EN**L**Q**L**TA**N**P **L**TL**INL**DS**Q**NL

**27 IRREKO L**KS**L**T**L**GG**S**Q **I**KE**INL**SNKKY

**28 IRREKO L**RS**I**Q**L**TS**N**K **I**SQ**IDL**TG**L**DA

**29 LRR L**EY**L**R**L**TD**N**P LTNVTKDYLDS

**C-TERM** LNGINGLKIYY

(G) *Listeria monocytogenes* lmo0331 protein

**SIGNAL** MKLSKILTIIVLSATVTSSL

**N-TERM** PIPMVKAESTTANEMKTENQLLKTDLKETPKEKTPNNNLKNQLVQ

AGTKTYNDYFPDDNLAKEVAETMNKNADESVTVEELAK

### Cons. LxxLxCxxNxLxxLDLxxNxx

### L

**1 SDS22 V**TK**L**D**A**RSQG**I**EDSTG**I**EY**L**TG

**2 IRREKO L**EV**L**N**L**ED**N**Q**L**KS**IDV**SK**N**LN

**3 IRREKO L**KE**L**T**C**SN**N**P**L**AN**LDV**SK**N**LA

**4 IRREKO L**EE**L**T**C**EN**N**E**L**TQ**LDV**SQ**N**TA

**5 IRREKO L**EY**L**Y**C**PR**N**Q**L**TK**LDV**SK**N**SA

**6 IRREKO L**RY**L**A**C**DV**N**Q**L**TN**LDV**SK**N**PA

**7 IRREKO L**TN**L**G**C**TK**N**Q**L**TD**LDV**SQ**N**PN

**8 IRREKO L**GT**L**V**C**SD**N**Q**L**TN**LDV**SQ**N**QA

**9 IRREKO L**EN**L**A**C**DN**N**E**L**KN**ID**INQALS

**10 IRREKO L**KE**L**S**C**EN**N**Q**L**TN**LD**TTQ**N**LA

**11 IRREKO L**EI**L**Y**C**DD**N**Q**L**TD**LDV**RKNVN

**12 IRREKO L**LI**L**F**C**NN**N**Q**L**TN**L**A**V**GET

**13 LRR I**AK**V**R**C**NN**N**Q**L**KDVSSLPDYF

**C-TERM** TDDNDDYQAMDQTLVSPTQTTQNNTLVYAVPTDLLDKDGNIVSII

KPDNGGIYDAATRTITWENLPDNGEVSYTFENEDYGRFSGRVTVP

YTGKETISISSDDEISYKEGTTKTEAAFLADIHASVTPATETITS

NFADVVDFQTPGKYVVTLSVAGSDVTKDVIVYVTAEPSEDNPVAP

TPPKDKDDTDVNNEQSPGNDKDGTDVNSGKSTDKQPVKVVEKQLP

KTGDITSLSLSLAGIVCLSFGILFFIKRKKKTV

(H) *Treponema denticola* TDE_0593

**SIGNAL** MKKFLTVLFLTGLLTTRIAAA

**LRRNT** ENPAKTAGSGRAILGISDDQKEIVVTAVTADGSAVHVEG**C**TVTEL

PSGEETILTATGAKVILKGA

### Cons. LxxLxCxxNxLxxLDLxxLxx

**1 IRREKO I**TK**L**D**C**GG**N**R**L**TE**LNV**QG**L**TA

**2 IRREKO L**QK**L**F**C**DD**N**L**L**TS**LDV**SG**V**TA

**3 IRREKO L**QS**L**S**C**GE**N**L**L**TS**LDV**SG**L**TG

**4 IRREKO L**RE**L**Y**C**NR**N**H**L**SS**LDV**QS**L**TA

**5 IRREKO L**QD**L**F**C**NA**N**K**L**TS**LNV**QD**L**KV

**6 IRREKO L**QR**L**H**C**NS**N**R**L**TL**LNV**RD**L**SA

**7 IRREKO L**QE**L**D**C**VG**N**E**L**TS**LDV**HG**V**TA

**8 IRREKO L**WE**L**E**C**SK**N**M**L**TL**LDV**QS**L**TS

**9 IRREKO L**SK**L**D**C**SA**N**Q**L**TS**LDV**RN**L**AA

**10 IRREKO L**EE**L**D**C**SN**N**K**L**TA**L**Y**V**QG**L**NA

**11 IRREKO L**QE**L**N**C**SE**N**E**L**TS**L**E**I**QG**L**TA

**12 IRREKO L**EV**L**DSGR**N**D**L**TS**LDV**QG**L**PA

**13 IRREKO L**KI**L**S**C**TV**N**E**L**TS**L**K**V**RD**L**PA

**14 IRREKO L**EK**L**D**C**SV**N**Q**L**TS**IDI**LE**L**TA

**15 IRREKO L**KE**L**N**C**SL**N**Q**F**TS**INI**LK**L**TA

**16 IRREKO L**KE**L**D**C**ST**N**Q**L**TS**LDV**RN**L**AA

**17 IRREKO L**EK**L**D**C**RD**N**K**L**TS**LNV**QG**L**NT

**18 IRREKO L**QK**L**Y**C**SE**N**E**L**TS**L**E**I**QG**L**KT

**19 IRREKO L**QK**L**N**C**YK**N**K**L**TS**LNV**QG**L**TA

**20 IRREKO L**QW**L**N**C**GY**N**E**L**TT**LNL**KG**L**HA

**21 IRREKO L**RD**L**E**C**FN**N**N**L**PE**LDV**QD**I**NT

**22 IRREKO L**QR**L**N**C**YH**N**K**L**ST**L**E**L**ST**L**HG

**23 LRR L**QE**L**C**C**YD**N**L**F**NEKTLIRILT

**C-TERM**ALPDRKQKKEGRALIYGKKNDLREGTITDFSSSAELKAAFEAAKA

　 KNWRFYKRDTVGNEEEV

(I) *Polaromonas naphthalenivorans* Pnap_3264

**N-TERM** MFPEVPYQLQMAPGCCHVLRCRPHHPKARENPLMADKERNVLIPL

PAGGLENIGSGPKSILSGMVSDALALARMREKSLAEKRFRIGDYE

FRDPDYRQILIWAKALEIEPEVFIRSFEDDSFSQDGNVLRLDVED

GSIVLLQWDFDIFPISSFEW

### Cons. LxxLxCxxNxLxx LDLxxLxx

**1 LRR V**DG**L**R**I**QQLG**F**KGIASAELSLRLPL

**2 IRREKO L**NC**L**D**C**HDIG**L**LTK**LDL**SN**L**PE

**3 IRREKO L**TY**L**E**C**GD**N**Q**L**VE **LDL**LN**V**PG

**4 IRREKO L**TD**I**Y**C**SN**N**Q**L**VK **LDL**SN**V**PK

**5 IRREKO L**TQ**L**L**C**EN**N**Q**L**TQ **LDL**SN**V**LG

**6 IRREKO L**TL**L**W**C**SS**N**S**L**IE **LNL**SN**V**QK

**7 IRREKO L**TD**L**W**C**ST**N**Q**L**TE **LDL**SD**V**SG

**8 IRREKO L**KE**L**H**C**NE**N**Q**L**VE **LDL**SN**V**PE

**9 IRREKO L**TE**L**N**C**DN**N**Q**L**TE **LDL**SN**V**SK

**10 IRREKO L**KH**L**R**C**QK**N**Q**L**AK **LDL**SN**V**PG

**11 IRREKO L**TG**L**D**C**SN**N**Q**L**VK **LDL**AN**V**QR

**12 IRREKO L**IG**I**F**C**GK**N**Q**L**AE **LDL**SN**V**PG

**13 IRREKO L**TT**L**W**C**RS**N**P**L**AE **LDI**RW**L**VK

**14 LRR L**KR**F**A**C**DPSVTLQKLPTQNF

(J) *Ddelta proteobacterium MLMS-1* MldDRAFT_4836

**N-TERM** MKEDKALIPRPNTGLATTQPGGNQIISRMTKGLLAQDRVNSLSQA

RFRIGDYELREPDYRQIMLWAEGLEMDAEELLVALEDSAHQIGVD

EISDYEDDVSTNNIFETVGFQLRDGQILSLSWDFDLLPRFINEWV

PGLALERLAFSSDGYYWIPPHILTFKLPSLRELVYVNIDQENLSL

NPHD

### Cons. LxxLxCxxNxLx xLDLxxLxx

**1 IRREKO L**PR**L**KLLYYFGPIE**LNL**TS**M**PE

**2 IRREKO L**ER**L**KYGGTEQREE**LDL**SP**V**PK

**3 IRREKO L**TW**L**D**C**SA**S**R**L** SA**LDL**TP**V**PG

**4 IRREKO L**TD**L**F**C**MRQS**L** KE**LDL**TP**V**PR

**5 IRREKO L**FQ**L**F**C**MD**N**Q**L** DK**LDL**TP**V**PG

**6 IRREKO L**VR**L**E**C**SR**N**Q**L** SE**LDL**TP**V**PG

**7 IRREKO L**IQ**L**F**C**LG**N**Q**I** GE**LDL**TP**V**TG

**8 IRREKO L**TM**L**D**C**SY**N**P**L** SE**LDL**TP**V**PR

**9 IRREKO L**TI**L**E**C**ND**N**Q**L** SE**LDL**TQ**V**PR

**10 IRREKO L**TI**L**E**C**KN**N**Q**L** SE**LDL**TP**V**PG

**11 IRREKO L**TM**L**F**C**WG**N**Q**L** SE**LDL**TP**V**PG

**12 LRR L**TM**L**N**C**DK**S**L**V**FHNAPPGLKINRR

(K) *Kordia algicida OT-1* KAOT1_04155

**SIGNAL** MKNVLFTVICLFVLSILNAQTTA

**N-TERM**  IPDANFEQALIDFGYDIVLDGEVFTDQIND

### Cons. LxxLxCxxNxLxxLDLxxNxx

**L L**

**1 SDS22 V**TE**L**Y**V**SNRN**I**SD**L**TG**I**ED**F**DS

**2 IRREKO L**VV**L**D**A**SQ**N**N**L**TS**LNI**NG**L**TA

**3 IRREKO L**QV**L**V**V**YQ**N**N**L**TS**IN**TAN**L**LQ

**4 IRREKO L**RQ**V**D**I**SN**N**N**F**ST**I**S**F**LN**N**VS

**5 IRREKO L**LT**L**D**C**HN**N**N**A**TS**I**T**L**NN**N**SN

**6 IRREKO L**EE**L**Y**A**FN**N**N**F**ST**INL**SN**N**QN

**7 IRREKO L**FV**V**D**L**AN**N**N**L**TE**LDV**LI**N**SQ

**8 IRREKO L**EY**L**S**V**YS**N**Q**L**TS**LNL**VNQSN

**9 IRREKO L**QE**L**V**C**YN**N**Q**L**TS**LDL**SA**N**TA

**10 IRREKO L**AA**L**Y**C**QT**N**Q**L**ES**L**S**I**KNGNN

**11 LRR I**NMSN**A**NF**N**A**L**NNPNLGCIQV

**ISLAND**  DDAANTYTNVDENAIFLEDCSLFGQDIYVPDDNFEQALIDLGIDT

SGVLDDYVAFADVIN

**12 SDS22 V**MN**L**D**V**SSKN**I**AD**L**TG**I**EA**F**FN

**13 IRREKO L**KT**L**D**V**TN**N**N**L**TA**LNI**NA**S**TN

**14 IRREKO L**EE**L**Y**C**AT**N**Q**L**TN**LDV**TS**N**VQ

**15 IRREKO L**QK**L**I**C**TE**N**Q**L**TS**IN**TNN**N**VW

**16 IRREKO L**EE**L**N**F**RD**N**Q**V**AS**IDL**SN**N**VL

**17 IRREKO L**KN**L**Y**I**TS**N**Q**L**TT**LDL**NN**N**TQ

**18 IRREKO L**EI**I**W**C**GD**N**L**L**TD**L**S**F**PNHPM

**19 IRREKO L**RD**C**F**V**TYGQ**L**ET**LDV**ASCAA

**20 IRREKO L**ED**L**R**C**FS**N**N**L**TE**LDV**SN**N**TQ

**21 IRREKO L**TF**L**R**A**NT**N**N**I**SC**I**A**V**AD**V**AY

**ISLAND**  AE ANWIGMVDNDVVFRNNCGPVYVPNDNFEQALIDLGYDNVLDDY

VDRMTAES

**22 SDS22 V**TT**L**N**L**SSKS**I**SD**L**TG**I**EA**F**LG

**23 IRREKO L**QF**L**N**V**TN**N**N**L**ST**IDI**SA**N**TE

**24 IRREKO L**KR**F**YGTL**N**Q**L**TS**LDV**SQ**N**AA

**25 IRREKO L**EH**L**Y**C**AQ**N**Q**L**SS**IDV**ST**N**TL

**26 IRREKO L**EE**L**G**V**AG**N**Q**L**AT**INI**SN**N**TA

**27 IRREKO L**EL**F**T**C**AN**N**Q**L**TS**LDV**SS**N**TA

**28 IRREKO L**DF**L**N**F**RN**N**Q**L**TN**IDL**ST**N**VL

**29 IRREKO L**TE**L**L**C**SD**N**Q**L**TA**LDV**SS**N**NL

**30 IRREKO L**ER**V**E**C**QT**N**Q**L**TS**LDF**NNHPF

**31 IRREKO L**ED**L**N**C**RE**N**L**L**TT**INI**NS**A**SA

**32 IRREKO L**KQ**V**R**I**DN**N**N**L**SQ**LNV**EL**N**TL

**33 IRREKO L**EN**L**S**C**SN**N**N**I**NS**LDL**SNQTA

**34 IRREKO L**IT**L**E**C**SD**N**N**I**TS**LDV**TN**S**NA

**35 IRREKO L**QT**L**N**C**SN**N**N**L**AV**LDV**AN**L**N

**36 IRREKO I**LD**L**N**C**SN**N**Q**I**NT**I**F**F**GN**M**SS

**37 IRREKO L**TN**F**D**C**SA**N**Q**L**SE**LDI**SQQAQ

**38 IRREKO L**VN**L**E**A**FN**N**L**L**SC**I**Q**V**SDESY

**ISLAND** ANLNWTTNVDTNVTFSLDCIDVLYINTDDFTTMALLALGTIDTNF

DGFISFEEAAAV

**39 SDS22** TGT**L**DLNNRG**I**TS**V**EG**L**NA**F**IN

**40 SDS22 　 I**TT**L**DISG**N**S**I**DD**L**SP**L**TG**L**SI

**C-TERM** DIVSRNSGASSEMSVTPMALEHLFADGNTFSTANLDDLTNLKTVD

LGNNLNLITVSIRNGNNAAITSFNTIGSTQLTCILVDDENAGYLM

TWSVEGTSNFTNTEAACLAILSTPETTLEEAFSIYPNPVSDNLTI

KTSTTLDTIEIYNASGQRIIKTNHTIINMANYPTGLYFVKLYSEK

KVIIKKVVKR

(L) *Coprococcus eutactus ATCC 27759* COPEUT_03021

**SIGNAL** MRKGRKWLPAVLSVAMVLQPFAGIGSVTAMA

**N-TERM**  ADGVNIDDTFRNDSIFKKYVSNNFDTNSDGYLDADEISAVRSIDI

HDIDSKAEYGRKAVQNVDGIEVFTN

### Cons. LxxLxCxxNxL xxLDLxxNxx

**L L**

**1 IRREKO L**TE**I**N**C**SGQG**I** KN**MDI**QN**L**TK

**2 IRREKO L**EY**L**D**V**SN**N**M**M** SD**L**T**L**PVAADD

**3 LRR L**AY**L**D**I**SG**N**D**I** TRLTSLSNYDN

**4 IRREKO L**TH**L**D**A**SG**T**N**L** ES**VDV**RS**M**KG

**5 IRREKO L**TY**L**S**V**SGLS**L** KT**LDL**SE**N**MQ

**6 IRREKO L**KT**L**R**C**QAMSGL ST**LDV**SDHTA

**7 LRR L**ES**L**Y**C**DA**T**ASSIKGSITKLDVSGDTS

**8 IRREKO L**IS**L**N**C**AS**N**N**L** AD**LDI**TD**T**PN

**9 IRREKO L**TI**L**D**C**SE**T**R**L** QS**LDI**AN**N**SK

**11 IRREKO L**TS**L**T**V**DG**T**P**L** GT**LDI**SS**N**TA

**12 IRREKO L**ST**L**S**A**SGIG**L** QD**VNI**SS**N**AA

**13 IRREKO L**KY**V**N**L**NS**N**D**L** KS**IDI**SS**N**AA

**14 IRREKO L**ET**L**Y**L**SD**N**I**L** ES**IDL**SR**N**VN

**15 IRREKO L**RD**L**T**L**DR**N**R**L** VC**IDV**SDCTK　-405

(M) *Clostridiales bacterium 1_7_47_FAA* Cbac1_010100006401

**SIGNAL** MKNTKYARGISLLLCTAMLAGQMGMTVYA

**N-TERM**  EEKSSSNLVGGGVCEHHPEHTEECGYVEAVKGHRCEHVHTDDCYT

DELICGYDDEDMDLATDSSATHVHKKKCYELDCPHKRGEHDDDCG

YIEAVKGHPCGFICDVCGKENPDADSGNVLPEIPEPEDKHPGTEV

PNQIPNQQEKVEALTITDFDVLDEKVQFQTVAPGTKLDELNLPAT

LGASGYTIEEDSTPAPEPITIKGITWEPDEAYDDTAEQGSYIFTP

VLPDGYTCAKDVELPEIYVRIGDANVTLANDINKYNNDDVRTFQA

ILDAHPSLVSGNVKKDNPNSWAGLVTWDESNPKR

### Cons. LxxLxCxxNxLx.xLDLxxLxx

### N

**1 IRREKO I**TK**L**VLDSQH**L**NGT**L**E**V**SG**L**TA

**2 IRREKO L**TN**L**S**C**SD**N**Q**L** TA**LDV**TGTA

**3 SDS22 L**TD**L**Y**C**SE**N**N**L** KT**L**SG**L**GN**L**TK

**4 IRREKO L**AY**L**Y**C**SE**N**Q**L** TT**LDV**SGTD

**5 SDS22 L**IV**L**N**C**YN**N**N**L** ET**L**DG**L**GS**L**MQ

**6 IRREKO L**TF**L**S**C**YN**N**Q**L** TA**LDV**SK**N**TQ

**7 IRREKO L**FG**L**S**C**YN**N**P**F** AS**F**K**I**KDDTI

**ISLAND** LTFNQTPGGTVETTSFTLSSSQVTLTAKPDIGYSFKEWTTLPSGV

SSSTNTVSINLTRSGTVEAAFVVDNPDDVNNDGYHDGDVAVINAI

IENNDLSAAKDDPAGWETDDLVRWDSSTPKR

**8 IRREKO I**TH**V**RIYDKG**L**RGT**LDV**SA**L**TN

**9 IRREKO L**DS**L**F**C**GK**N**N**L** TG**LNV**SK**N**TS

**10 SDS22**  **L**TY**L**S**C**ID**N**Q**L** EG**M**LD**V**SG**L**TE

**11 SDS22**  **L**MT**F**D**C**YN**N**Q**L** TA**L**NG**L**ES**L**EN

**12 IRREKO L**TS**L**Y**C**YN**N**Q**L** KA**LDV**SS**L**SS

**13 IRREKO L**HT**L**S**C**FD**N**R**L** QR**LNV**SG**L**TS

**14 IRREKO L**TL**L**F**C**DN**N**Q**L**TGA**LDV**SG**S**PL

**15 IRREKO L**ER**L**Y**C**NN**N**Q**L** TE**LNL**SS**L**TS

**16 IRREKO L**TD**L**H**C**MNLP**L** TY**F**T**A**PDGSL

**C-TERM** LEIKPGSGGKIILGDIKNPGIEGYYLDRKEVILSAVPDTGYRFTG

WTRDGAEAGNSLVLGFILSGNSTVTANFEEIITRGGSSSSGGGGS

IITVPTIKWIRDERGWRLKNPDQTWAISSWKEVNGIWYHFNEEGY

MQTGWYTDIDGNQYYLLPTDGSTQGSMVTGWQLIDNKWYYFNMES

DGTKGRLLYNTVTPDGYYVNEKGEWIE

(N) 　 *Listeria* lin1204/LMOf6854_0364

**N-TERM**  MKSKAKYIIIVGVVLFQSLVAYPLITMAEENDSKSVNIETTLEPK

EVESTTSETEMEASTKEVVEEKTSQKSIITNNLTQEKSVLQAGET

YESTFPDAALATVIAKAATGSEDITQEVSQTDLNK

### Cons. LxxLxCxxNxL xxLDLxxxxx

**L**

**1 SDS22 I**TS**L**T**A**TSKG**I** VD**L**TG**I**NL**L**SK

**2 SDS22 L**AY**L**S**M**SG**N**Q**I** SD**I**SA**L**NG**L**AN

**3 LRR L**SN**L**N**V**SS**N**Q**I** TSFNLNANSNLPM

**4 IRREKO L**NT**V**D**I**RS**N**DL NN**INV**QDQSK

**5 LRR L**RT**I**K**C**DTGSSSEL TEVMLKNLPT

**6 LRR L**IA**A**SNSS**N**PV ADDIAFSSTPGLSKVILENLPST

**7 IRREKO** SSS**V**Q**L**DR**C**V**I** EE**L**V**I**NNLPK

**8 SDS22 V**SI**V**T**I**NN**N**K**I** TT**L**EG**L**ED**L**AA

**9 SDS22 L**TS**L**N**A**GN**N**E**L** TE**I**EN**M**HT**F**PS

**10 LRR L**QT**L**N**L**SS**N**D**L** TNMVMNQATAEKFPL

**11 IRREKO L**RT**M**D**I**RS**N**N**L** IK**IDI**QNQSK

**12 LRR L**AT**I**I**C**DTGSSSEL IEVTLKNLPE

**13 LRR L**IA**A**SNGS**N**Q**V** KDDIAFLSTPGLSKVILENLPST

**14 IRREKO** SSS**V**Q**L**DR**C**V**I** EE**L**V**I**NNLPK

**15 SDS22 V**SI**V**T**I**NN**N**K**I** TT**L**EG**L**ED**L**AA

**16 SDS22** LTS**L**N**A**GN**N**E**L** TE**I**ES**M**HA**F**PK

**17 LRR L**QT**L**T**L**IS**N**K**L** TSIILNQATANGTPN

**18 IRREKO L**TS**I**DSRS**N**D**L** TK**IDI**QDQPK

**19 LRR L**TK**I**I**C**DTGSSAEL TELTLRNLPE

**20 LRR L**IA**A**SNGS**N**A**V** SDDIAFSATPGLSKVTLENLLAT

**21 IRREKO** NVT**I**Q**L**DH**C**V**I** EE**L**V**I**NNVPQ

**22 SDS22 V**AF**I**Y**I**NN**N**K**I** TT**L**EG**L**GN**L**TA

**23 SDS22 V**TN**L**N**A**EN**N**E**L** TE**L**EN**I**PA**F**PK

**24 LRR L**KT**L**T**V**NN**N**H**I** SVLPTSLKTKNPV

**25 LRR L**TT**L**S**A**TNQT**I** TLKQKVIVSDL

**C-TERM** VLDNEVKNFGQTTTAKSISNNGTYQNNQVTWLFEDVQGVNAVDY

QFSEPIQEATIQGTFSGKVTQPIKVSTVPVITADTEMTYPKNAT

ISEAAFFKDISASVTDDAILTSDFESVVDFVKAGTYEVTLNAMN

EDGVEAVSVTVLVHIAKSPAPVITADKEITYTQSTEVSTTEFLV

AIHATTNDGSPIESDLDTTVNWSKVGDYTITLSATNEDGVEAI P VKVAVHIVEPPAPTISNVIFDVDGVQTTESHEVGELVSEPLTPT

KEGYTFIGWYDEKTGGNKWDFTTDKMPGYNITLYAQFSKDTNK G EDGDG GKASVASSMEVTPSGQSQSENSKNSSNIKLPATGDDNAT

VLLVGL GLLMLGLFIRLTQKKHTK

(O) *Escherichia coli SMS-3-5* EcSMS35_1703

**LRRNT** MTNINTA**C**VKNNASYQLNNALPNKETISSNF**C**ERLAQWGNKSLNN

GEERAIAVERIKEAYNSN

## Cons. LxxLxLxxNxLxxLDLxxQxx

## N

**1 BAC M**AS**L**D**L**SYLD**L**SE**LP**P**IP**ST

**2 IRREKO V**NT**L**N**L**EN**N**C**L**TC**LDF**TD**N**AS

**3 IRREKO L**VN**I**N**L**SF**N**K**I**KT**I**T**F**PN**Q**SK

**4 IRREKO L**EN**I**Y**I**DH**N**N**L**EN**LDL**KN**Q**LS

**5 IRREKO L**VN**L**E**A**QN**N**N**L**TK**INI**SD**S**YK

**6 IRREKO L**KF**L**N**L**DY**N**K**L**AS**LDL**SR**Q**ES

**7 IRREKO L**IE**L**S**A**HH**N**M**I**ND**L**I**L**HNHPI

**8 IRREKO V**EK**I**T**L**ND**N**H**I**AH**LNA**KT**T**TK

**9 LRR L**EY**L**N**L**SN**N**N**L**LPTDDIDQLISS

**10 LRR** KHLWH**V**LV**N**G**I**NNDPLAQMQYWTAVRNIIDDTN

**11 LRR** EVT**I**D**L**SY**N**L**A**ITNIDTSDEH

**C-TERM** LVEVSENSEGNHIKENDSMSIRYRSKYYSREYALIEEETIFSDAE

LKAILPMRRMYGVGDYKSNSSSLPSHSGL KDPTGTPVCYYIHNED

KPSLGFGPTSNNWLSQSFTTEL

(P) *Escherichia coli O157:H7* ECS2075/Z2240

**LRRNT**  MTNINTA**C**VKNNASYQFNNALPNKETISSNF**C**ERLEQWGNKSLNN

GEERAIAVERIKEAYNSN

### Cons. LxxLxLxxNxLxxLDLxxxxx

**1 BAC** **M**ASLDLSYLD**L**SE**LP**P**IP**ST

**2 IRREKO V**NT**L**N**L**EN**N**C**L**TC**LDF**TDNAS

**3 IRREKO L**VN**I**N**L**SF**N**K**I**NT**I**T**F**PNESN

**4 IRREKO L**EN**I**Y**I**DH**N**N**L**ES**LDL**KNQHS

**5 IRREKO L**VN**L**E**A**QN**N**N**L**KK**L**I**F**LIVIN

(Q) *Trichomonas vaginalis G3* TVAG_084780

**N-TERM**  MAVTFNSLAMSEKPKLTPSMRGIEMA

### Cons. LxxLxLxxNxLxxLDLxxLxx

**1 LRR V**FE**L**D**I**SGKD**V** AEIQNEKPLDE

**2 LRR I**RK**L**T**A**SQ**N**Q**I** QSLAFLEHTPN

**3 SDS22 L**VD**L**D**L**SQ**N**Q**I** SEG**V**SN**F**SL**L**KF

**4 SDS22 I**HS**I**N**L**SS**N**L**F** EN **V**NG**F**PT**L**NT

**5 SDS22 L**TY**L**D**L**SS**N**H**L** AS **A**GD**I**PS**L**PF

**6 IRREKO L**KH**L**N**L**SN**N**S**I** TA**LNL**AV**M**PS

**7 IRREKO L**QI**L**N**L**QG**N**L**L** AK**L**E**L**PN**L**PS

**8 LRR I**RE**I**D**A**SH**N**S**I** ETIDQFTEESLPY

**9 LRR L**WS**L**N**L**RY**N**Q**L**KTPEELHSFEKLPL

**10 LRR L**FD**L**K**I**EN**N**P**L** IQEDKSHIPPI

**C-TERM**  LVILPTLTILDGEQVNAKNKVKAELSVKSETML
