# Supplementary material for: A nested leucine rich repeat (LRR) domain: The precursor of LRRs is a ten or eleven residue motif
Source: BMC Microbiol. 2010 Sep 9;10:235. doi: 10.1186/1471-2180-10-235 (PMC2946307; doi:10.1186/1471-2180-10-235)

(A) *Bifidobacterium animalis* BIFLAC_05879

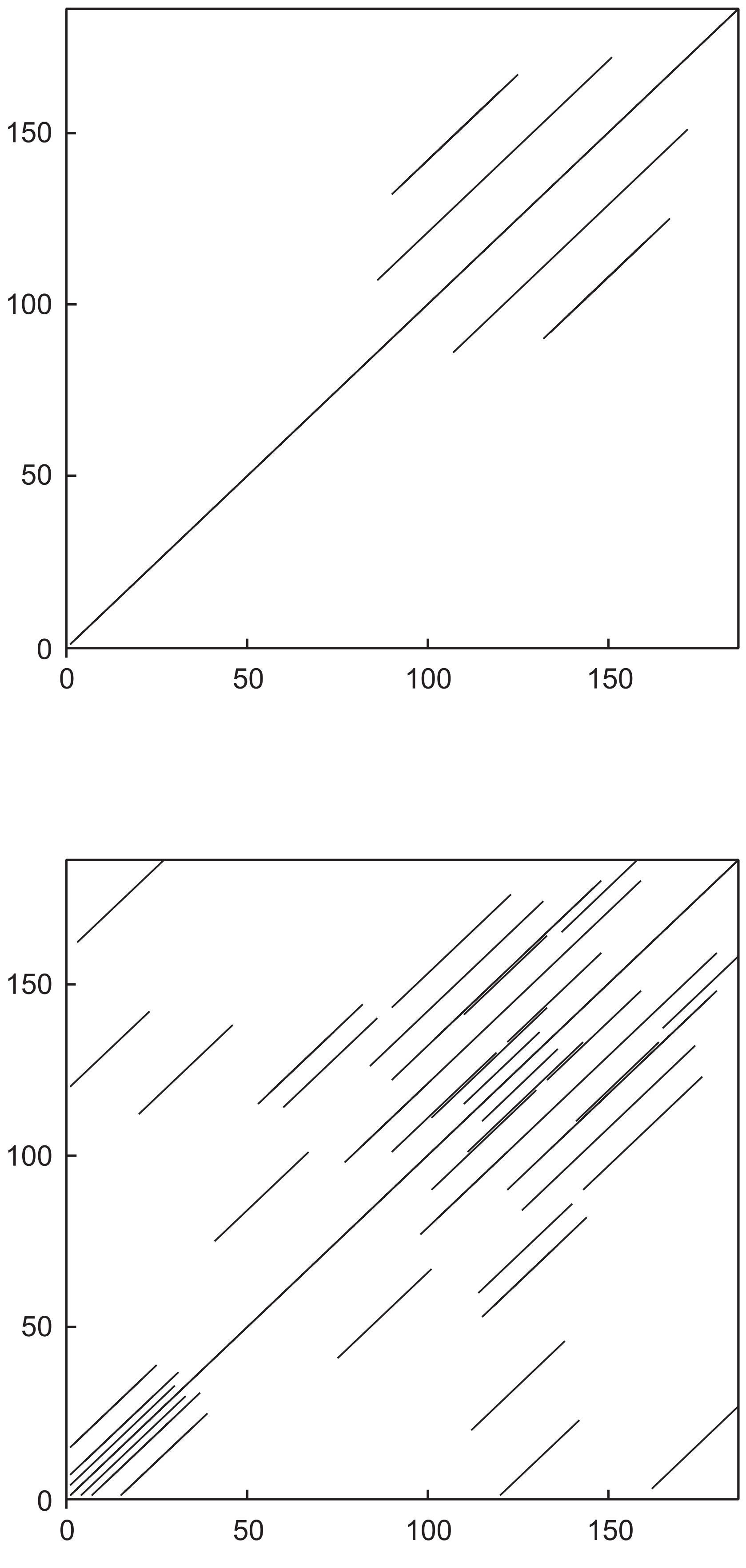

(B) *Vibrio harveyi HY01* AIQ_3393

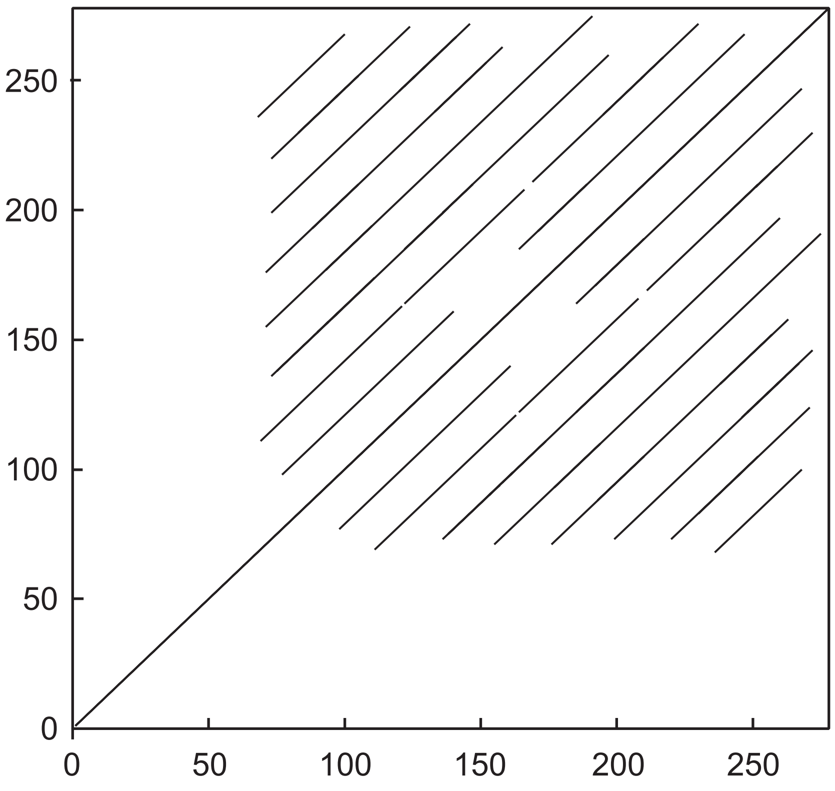

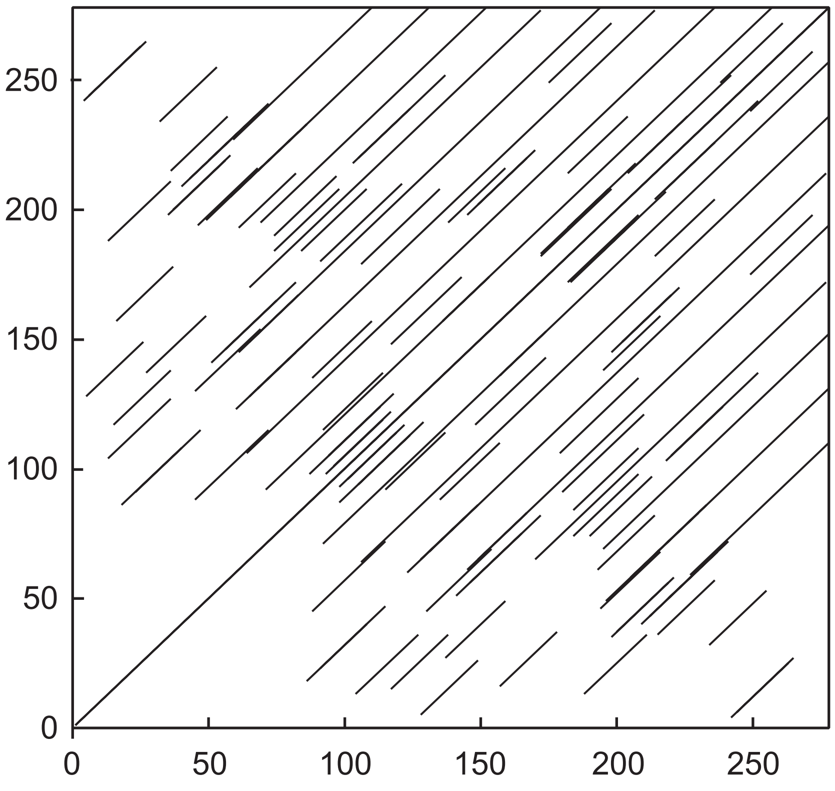

(C) *Listeria monocytogenes* lmo0331

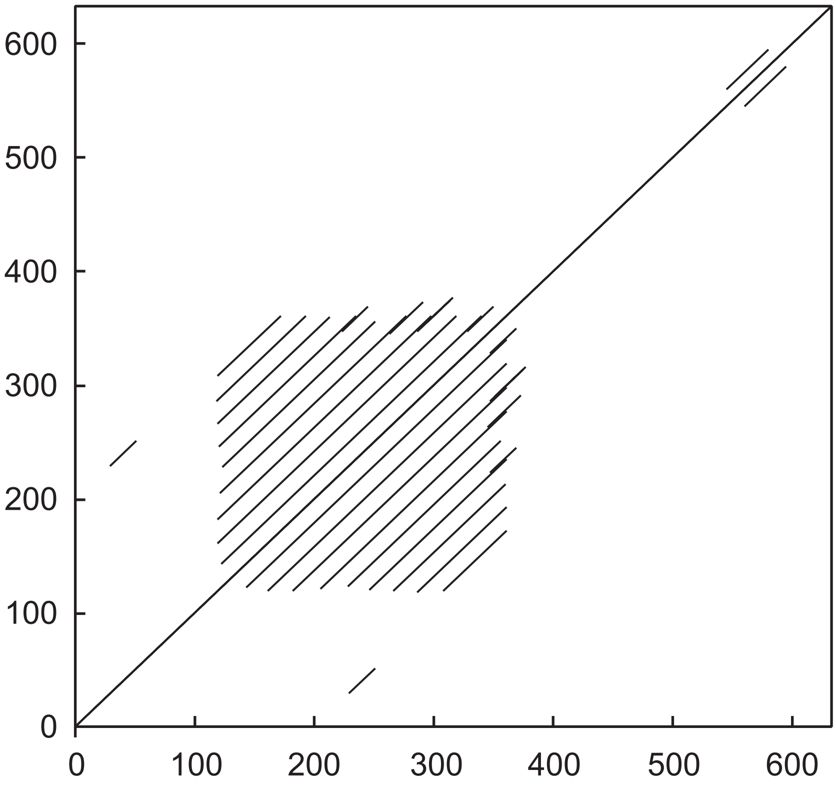

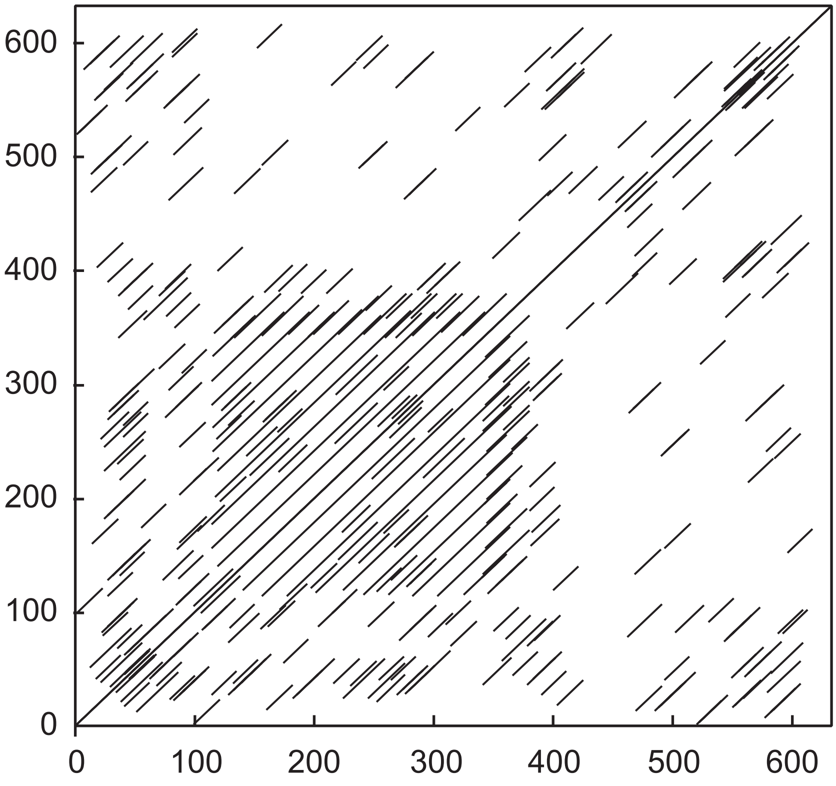

(D) *Treponema denticola* TDE0593

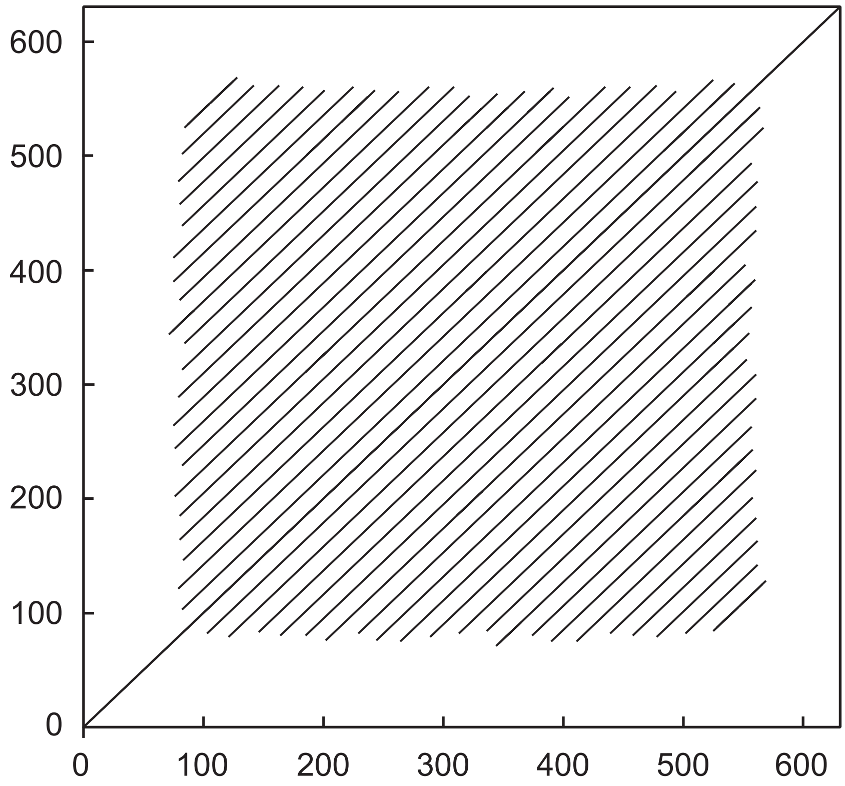

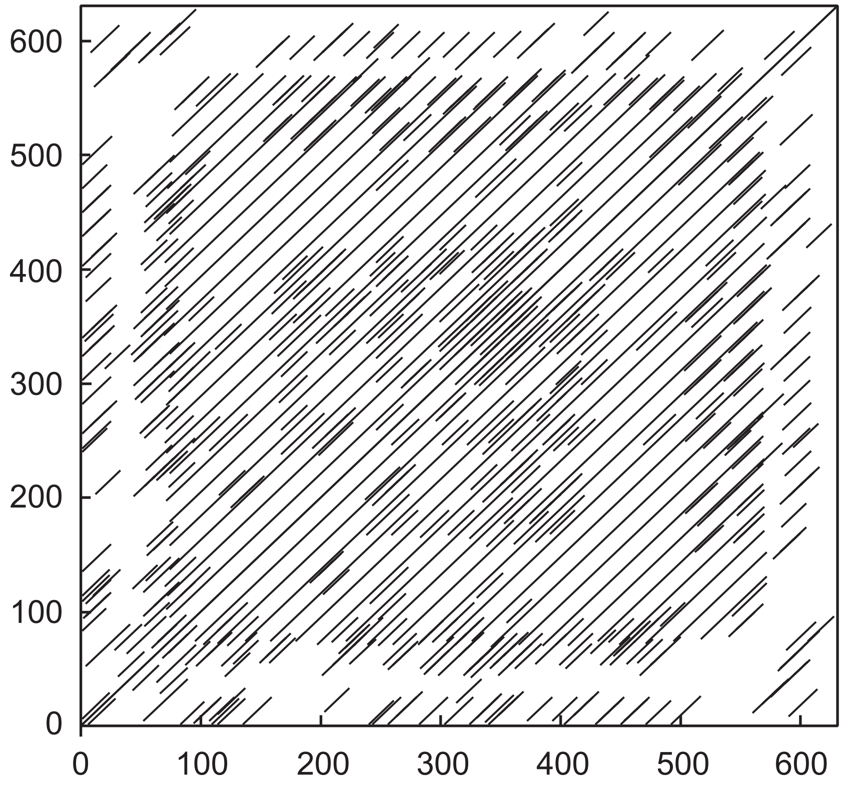

Supplement: Additional file 3 — Figure S2: Self-dot matrices for four IRREKO@LRR proteins. (A) Bifidobacterium animalis BIFLAC_05879; (B) Vibrio harveyi HY01A1Q_3393, (C) Listeria monocytogenes lmo0331 protein; (D) Treponema denticola TDE_0593. A window size of 21 residues was used. The threshold is 30 in the upper panel and 10 or 15 in the lower panel. Residues used are full lengths for the self-dot matrices; residue 1-186, 1-278, 1-633, and 1-631 of BIFLAC_05879, HY01A1Q_3393, lmo0331 protein, TDE_0593, respectively, were used. The abscissa and the ordinate are residues number. [file 1471-2180-10-235-S3.PDF]
