# Supplementary material for: A nested leucine rich repeat (LRR) domain: The precursor of LRRs is a ten or eleven residue motif
Source: BMC Microbiol. 2010 Sep 9;10:235. doi: 10.1186/1471-2180-10-235 (PMC2946307; doi:10.1186/1471-2180-10-235)
Supplement: Additional file 4 — Figure S3: Protein secondary structure prediction in five IRREKO@LRR proteins by the Proteus and SSpro4.0 programs. (A) Escherichia coli yddk; (B) Bifidobacterium animalis BIFLAC_05879; (C) Vibrio harveyi HY01 A1Q_3393; (D) Listeria monocytogenes lmo0331 protein; (E) Shewanella woodyi ATCC 51908 SwooDRAFT_0647; (F) Treponema denticola TDE_0593. The highly conserved segment of individual LRRs is highlighted by a shadow. For comparison, its consensus sequence is shown in bold letters. Abbreviations: h/H, helix; c/C, coil; e/E, β-strand. [file 1471-2180-10-235-S4.DOC]

(A)　 *Escherichia coli* yddk

**LxxLxLxxNxL LxxLxLxxNxL LxxL**

**Seq.** MITDLILHNHPRMKTITLNDNHIAHLNAKNTTKLEYLNLSNNNLLPTNDIDQLISSKHLW

**Proteus** ccccccccccccceeeeeccccccccccccccccceeecccccccccccccccccceeee

**SSpro** CCHHHHHCCCCCCEEEECCCCHHHHECCCCCCEEEEEECCCCCCCCCCCHHHHHCCCCEH

**xLxxNxL LxxLxLxxNxL LxxLxLxxNxL**

**Seq.** HVLVNGINNDPLAQMQYWTAVRNIIDDTNEVTIDLSGLNLTTQPPGLQNFTSINLDNNQF

**Proteus** ccccccccccccccccccccccccccccceeeeecccccccccccccccceeeeeccccc

**SSpro** EHEECCCCCCHHHHHHHHHHHHHHHCCCCEEEEECCCCCCCCCCCCCCCCCEEECCCCCC

**LxxLxLxxNxL LxxLxLxxNxL LxxLxL**

**Seq.** THFDATNYDRLVKLSLNSNALESINFPQGRNVSITHISMNNNALRNIDIDRLSSVTYFSA

**Proteus** ccccccccccceeeeecccccccccccccccccceeeecccccccccccccccccceeec

**SSpro** CECCCCCHHHHEEEECCCCHHHHCCCCCCCCEEEEEEECCCHHHHCCCHHHHCCHHHHHH

**xxNxL LxxLxLxxNxL LxxLxLxxNxL Lxx**

**Seq.** AHNQLEFVQLESCEWLQYLNLSHNQLTDIVAGNKNELLLLDLSHNKLTSLHNDLFPNLNT

**Proteus** cccccccccccccccceeeeeccccccccccccccccceeeecccccccccccccccccc

**SSpro** HHCHCHHHHHHHHHHHHHHHCCHHHHHHHHHCCHHHHHHHCCCHHHHHHHHHHHHHCHHH

**LxLxxNxL LxxLxLxxNxL LxxLxLxxNxL**

**Seq.** LLINNNLLSEIKIFYSNFCNVQTLNAANNQLKYINLDFLTYLPSIKSLRLDNNKITHIDT

**Proteus** eeccccccccccccccccccceeeecccccccccccchhhccccceeeeccccccccccc

**SSpro** HHHHHHHHHHHHHHHHCHCCHHHHHHHHHHHHHHCHHHHHHCCCCCEEECCCCCEEEEEC

**Seq.** NNTSDIGTLFPIIKQSKT

**Proteus** hhhhhhhhhhhhhhhhcc

**SSpro** CCCCCHHHHHHHECCCCC

(B) *Bifidobacterium animalis* BIFLAC_05879

**Seq.** MMNTLKKMAVSAVAALAGVMMVAPTAMAANGTIPIDAAHFPDTAFRVRIAQEFDSYPKDG

**Proteus** cchhhhhhhhhhhhhhhhhhhhhhhhhccccccccccccccchhhhhhhhhhhccccccc

**SSpro** CHHHHHHHHHHHHHHHHHHHHECHHHHHHCCCCCCCCCCCCCCCEEEEEHHHHCCCCCCC

**LxxLxLxxNxL LxxLxLx**

**Seq.** KLTIAERNAVTEIRGGDSYEIKFAKGIGYFPTLENLYLYNQLLTSIDLSHNAKLQNLNLS

**Proteus** eechhhcccccceecccccccccccchhhccceeeeeeccceeeeeccccccccceeeec

**SSpro** CEEEEEHCCCEEEECCCCCCHHHHCCCCCCCCHHHHHHHHHHHHHCCCCCCHHHHHCCCC

**xNxL LxxLxLxxNxL LxxLxLxxNxL**

**Seq.** RNKLTKIDLSRNPELRDLDLSSNKLISINVSKNTKLTEFDFQSNPLLWISSIAPAVNVDI

**Proteus** ceeecccccccccccceeeeccceeeeeccccccceeeeeeccccccccccchhhhhhhh

**SSpro** CCCCCEEECCCCCCHHCCCCCCCCEEEEEECCCCEEEEEEECCCCCCHHHCCCCCECEEE

**Seq.** YSPFTD

**Proteus** hhhhcc

**SSpro** ECCCCC

(C) *Vibrio harveyi HY01* A1Q_3393

**LxxLxLx**

**Seq.** MSRNKQKLVMATCGFLVSLNAFSGEVLDVSDIKFEDDNFQKCVLAQKVTNPTEITKLVCR

**Proteus** ccccceeeeecccceeeeeeccccccccccccceecccccccccccccccccceeeeecc

**SSpro** CCCCHHHEEHHHHHHHEEHHHHCCCEEEHHHCCCCCHHHHHHHHHHHHHCCCCCCEEEEC

**xNxL LxxLxLxxNxL LxxLxLxxNxL Lxx**

**Seq.** QFNIDSANEIHYFPALKELWLSGKQLSNIDLSKNLELTSISITKSNLTSLDLSNNPNLTE

**Proteus** ccccccccccccccccceeeecccccccccccccccceeeeecccccccccccccceeee

**SSpro** CCCCCCCCCCCECCCCEEEEECCCCCCCCCHHHCCCCEEEEECCCCCCCEECCCCCCCEE

# LxLxxNxL LxxLxLxxNxL LxxLxLxxNxL

**Seq.** LNVSFNQISELDLTKSPKITDLSVVSNQIEALNLSSNIELKYLWAKENKLKDIDFSHNPN

**Proteus** eeccccccccccccccccceeeeeccceeccccccccccccceecccccccccccccccc

**SSpro** EECCCCCCCEEECCCCCCCCCCEEECCCCCECCCCCCCCCEEEEECCCCCEEEECCCCCC

**LxxLxLxxNxL LxxLxLxxNxL LxxLxLxxNxL**

**Seq.** LVQISLDNNQLSKVNISNNEQLNKLSLMFNLLSSLDVSKNVKLTKLYVTDNKLTKLDVSH

**Proteus** ceeeeeccceeccccccccccceeeecccceecccccccccccceeeeccceeeeccccc

**SSpro** CEEEEECCCCEEEEEECCCCCCCEEECCCCCCCCCCCCCCCCCEEEECCCCCEEEEEECC

**LxxLxLxxNxL**

**Seq.** NPKLRALWATENPLEDLDITSNSSLSDYEVDEGVLVQE

**Proteus** ccccceeeeccccccccccccccccceeeecccccccc

**SSpro** CCCEEEEEECCCCCCEEEECCCCCEEEEEECCCCEECC

(D) *Shewanella woodyi ATCC 51908*　 SwooDRAFT_0647

**Seq.** MDYKQPLAMTLLSLAVSSSCFAADYQNVKDIPFKDSNFKACVLAQTTEDPAAITKLICRQ

**Proteus** cccccccceeeeeeeccccccccccccccceeecccccccccccccccccccceeeeeec

**SSpro** CCCCCHHHHHHHCCCCCCCCEEECCCCCCCCCCCCHHHHHHHHHCCHHHHHHHHCCCCCC

**LxxLxLxxNxL LxxLxLxxNxL LxxL**

**Seq.** MKINQVDELSHFPALERLWISGPQLKEVDLSGNLKLTELSITKGKLTQLDLSKNSQLQEL

**Proteus** ccccccccccccccceeeeecccccccccccccccceeeeecccccceeeccccccceee

**SSpro** CCCCCHHHHHHCCCCEEEEECCCCCCEEECCCCCCCCEEEECCCCCCECCCCCCCCCEEE

**xLxxNxL LxxLxLxxNxL LxxLxLxxNxL L**

**Seq.** NISFNQISDLDLTHNPKLTDISVVANRLTELDLSQNSALKYLWAKENQFTSLDFSHNSAL

**Proteus** eeccceeeeeecccccccceeeeccccccceeecccccccceeccccccccccccccccc

**SSpro** EECCCCCCEEECCCCCCCEEEEECCCCCCECCCCCCCCHHEECCCCCCCCECCCCCCCCC

**xxLxLxxNxL LxxLxLxxNxL LxxLxLxxNxL**

**Seq.** VQVGLTSNQISTIDVSKNSELNRLGLMYNQLTKLDVSYNSKLTKLYLTDNKLTHIDISQN

**Proteus** ceeeecccccceeeccccccccceeccccccccccccccccccceecccccccccccccc

**SSpro** CEEECCCCCCCECCCCCCCCCEEEECCCCCEEEEECCCCCCCEEEEECCCCCCEEECCCC

**LxxLxLxxNxL**

**Seq.** PKLRDLWATGNPLEQLDISKNPKLDDYEVDEGIKISE

**Proteus** cccceeeeccceeeeeeccccccccceeeccceeecc

**SSpro** CCCEEEECCCCCCCEEECCCCCCCEEECCCCCEEECC

(E) *Listeria monocytogenes* lmo0331 protein

**Seq.** MKLSKILTIIVLSATVTSSLPIPMVKAESTTANEMKTENQLLKTDLKETPKEKTPNNNLK

**Proteus** cccccceeeeeecccccccccccccccccccccccccccceeeeeccccccccccchhhh

**SSpro** CCCEEEEEEEEEEEEEECCCCCCEEEEEEHCCCCCCCCCCEECCCCCCCCCCCCCCCCHH

**LxxLxCxxNxL**

**Seq.** NQLVQAGTKTYNDYFPDDNLAKEVAETMNKNADESVTVEELAKVTKLDARSQGIEDSTGI

**Proteus** hhhhhhcccccccccccchhhhhhhHhhccccccccccccccceeeeeeccccccccccc

**SSpro** CCCCCCCCCCHHHCCCCHHHHHHHHHHHCCCCCCCECHHHHHHHHEECCCCCCCCCHCHH

# LxxLxCxxNxL LxxLxCxxNxL LxxLxCxxNxL

**Seq.** EYLTGLEVLNLEDNQLKSIDVSKNLNLKELTCSNNPLANLDVSKNLALEELTCENNELTQ

**Proteus** cccccceeeeeccccccccccccccceeeeeeccceecccccccccccceeeeccccccc

**SSpro** HHHCCCEEEECCCCCCCCCCHCCCCCCHEEECCCCCCCCCCCCHHCCHHECCCCCCCCCE

**LxxLxCxxNxL LxxLxCxxNxL LxxLxCxxNx**

**Seq.** LDVSQNTALEYLYCPRNQLTKLDVSKNSALRYLACDVNQLTNLDVSKNPALTNLGCTKNQ

**Proteus** cccccccccceeeecccceeecccccccccceeeecccccccccccccccceeeeecccc

**SSpro** CCHCCCCCCEEEECCCCCCCCCCHCCCCCCEEEECCCCCCCECCCCCCHHHHHEECCCCC

**L LxxLxCxxNxL LxxLxCxxNxL LxxLxCx**

**Seq.** LTDLDVSQNPNLGTLVCSDNQLTNLDVSQNQALENLACDNNELKNIDINQALSLKELSCE

**Proteus** ccccccccccccceeeecccccccccccccccceeeecccccccccccccccccceeeec

**SSpro** CCCCCCCCCCCCEEEECCCCCCCECCHHHCCCCEEEECCCCCCCCCCCCCCCCHHHECCC

**xNxL LxxLxCxxNxL LxxLxCxxNxL LxxLxC**

**Seq.** NNQLTNLDTTQNLALEILYCDDNQLTDLDVRKNVNLLILFCNNNQLTNLAVGETIAKVRC

**Proteus** cccccccccccccceeeeecccceeeccccccccceeeeeCcccccccccccccccceee

**SSpro** CCCCCCCCHHHHHHHHHHECCCCCCCECCCCCCCCCEEEECCCCCCCCCCCCCCCCEEEC

**xxNxL**

**Seq.** NNNQLKDVSSLPDYFTDDNDDYQAMDQTLVSPTQTTQNNTLVYAVPTDLLDKDGNIVSII

**Proteus** ccccccccccchhhhhhhhhhhhcccccccccccccccceeeeeeccceeeccccccccc

**SSpro** CCCCCCCCCCCCCHCCCCCCEEEECCCEEECCCCCECCCEEEEECCCCCECCCCCEECCC

**Seq.** KPDNGGIYDAATRTITWENLPDNGEVSYTFENEDYGRFSGRVTVPYTGKETISISSDDEI

**SSpro** ccccceeeccccccccccccccceeeeeeeccccccccceeeeeeeeeeeeeeeeccccc

**SSpro** CCCCCCCCCCCCCCEECCCCCCCCEEEEEEECCCCCCCCCCEEEEEEEECCCEEEECCCE

**Seq.** SYKEGTTKTEAAFLADIHASVTPATETITSNFADVVDFQTPGKYVVTLSVAGSDVTKDVI

**Proteus** ccccceeeeeccccccccccccccccccceeeeeeeeeeccceeeecccccccccccccc

**SSpro** EECCCCEEEHHHHHHHCCCCCCCCCCCECCCCCCEEECCCCCCEEEEECCCCCCCCEEEE

**Seq.** VYVTAEPSEDNPVAPTPPKDKDDTDVNNEQSPGNDKDGTDVNSGKSTDKQPVKVVEKQLP

**Proteus** eeeeeeeccccccccccccccccccccccccccccccccccccccccccccceeeeeecc

**SSpro** EEEEECCCCCCCCCCCCCCCCCCCCCCCCCCCCCCCCCCCCCCCCCCCCCCCCCECCCCC

**Seq.** KTGDITSLSLSLAGIVCLSFGILFFIKRKKKTV

**Proteus** ccccccchhhhhhhhhhhhhhhhceeeeecccc

**SSpro** CCCCCCCCCHEHHHHHHHHHHHHHEHHCCCCCC

(F) *Treponema denticola* TDE_0593

**Seq.** MKKFLTVLFLTGLLTTRIAAAENPAKTAGSGRAILGISDDQKEIVVTAVTADGSAVHVEG

**Proteus** cccchhhhhhhhcccchhhhccccccccccccccccccchhhhhcccccccccceeeccc

**SSpro** CHHHHHHHHHHHHHHHHHHECCCCHHHHCCCEEEEEEECCCCCCEEEEEECCCCEEEEEE

**LxxLxCxxNxL LxxLxCxxNxL**

**Seq.** CTVTELPSGEETILTATGAKVILKGAITKLDCGGNRLTELNVQGLTALQKLFCDDNLLTS

**Proteus** ceeeeccccccccccccccccccccccceeeecccccccccccccccceeeecccccccc

**SSpro** EEEEEEECCCCCCCCCCCCHHHHCCCCEEEECCCCCCCCCCHHCCCCCEEEECCCCCCCC

**LxxLxCxxNxL LxxLxCxxNxL LxxLxCxxNx**

**Seq.** LDVSGVTALQSLSCGENLLTSLDVSGLTGLRELYCNRNHLSSLDVQSLTALQDLFCNANK

**Proteus** cccccccccceeeeccceecccccccccccceeeecccccccccccccccccceeccccc

**SSpro** CCHCCCCCCEEEECCCCCCCCCCCCCCCCCEEEEECCCCCCCCCHCHCCCHEEEECCCCC

**L LxxLxCxxNxL LxxLxCxxNxL LxxLxCx**

**Seq.** LTSLNVQDLKVLQRLHCNSNRLTLLNVRDLSALQELDCVGNELTSLDVHGVTALWELECS

**Proteus** cccccccccccccceeccccccccccccccccccceeccccccccccccccccccceecc

**SSpro** CCCCCCCCCCCCEEEEECCCCCCCCCCCCCCCCEEEECCCCCCCCCCCCCCCCHEEEECC

**xNxL LxxLxCxxNxL LxxLxCxxNxL LxxL**

**Seq.** KNMLTLLDVQSLTSLSKLDCSANQLTSLDVRNLAALEELDCSNNKLTALYVQGLNALQEL

**Proteus** ccccccccccccccccceecccccccccccccccccceeeccccccccccccccccccce

**SSpro** CCCCCCCCCCCCCCCEEEECCCCCCCCCCCCCCCCCEEEECCCCCCCCCCHCCHHHHHEE

**xCxxNxL LxxLxCxxNxL LxxLxCxxNxL L**

**Seq.** NCSENELTSLEIQGLTALEVLDSGRNDLTSLDVQGLPALKILSCTVNELTSLKVRDLPAL

**Proteus** eecccccceeccccccccccccccccccccccccccccceeccccccccccccccccccc

**SSpro** ECCCCCCCCCCCCCCCCCEEEECCCCCCCCCCCCCCCCHEEEECCCCCCCCCCCCCCCCC

**xxLxCxxNxL LxxLxCxxNxL LxxLxCxxNxL**

**Seq.** EKLDCSVNQLTSIDILELTALKELNCSLNQFTSINILKLTALKELDCSTNQLTSLDVRNL

**Proteus** cccccccccccccccccccccccccccccccccccccccccccccccccccccccccccc

**SSpro** CEEECCCCCCCCCCCCCCCCCEEEECCCCCCCCCCCCCCCCCEEEECCCCCCCCCCCCCC

**LxxLxCxxNxL LxxLxCxxNxL LxxLxCxxNxL**

**Seq.** AALEKLDCRDNKLTSLNVQGLNTLQKLYCSENELTSLEIQGLKTLQKLNCYKNKLTSLNV

**Proteus** ccceeeeeccccccccccccccccceeeecccccccccccccccccceeccccccccccc

**SSpro** CCCEEEECCCCCCCCCCCCCCCCHHEEECCCCCCCCCECCCCCCCEEEECCCCCCCCCEC

**LxxLxCxxNxL LxxLxCxxNxL LxxLxCxxNxL**

**Seq.** QGLTALQWLNCGYNELTTLNLKGLHALRDLECFNNNLPELDVQDINTLQRLNCYHNKLST

**Proteus** ccccccceeeeccccccccccccccccceeecccccccccccccccccceeecccccccc

**SSpro** CCCCCCEEEECCCCCCCCCCCCCCCCCEEEEECCCCCCCCCCCCCCCEEEEEECCCCCCC

**LxxLxCxxNxL**

**Seq.** LELSTLHGLQELCCYDNLFNEKTLIRILTALPDRKQKKEGRALIYGKKNDLREGTITDFS

**Proteus** cccccccccceeeecccccccccccccccccccccccccceeeeeccccccccccccccc

**SSpro** CCHCCCCCCEEEEECCCCCCCCCHHHHHHHCCCCCHHHHHHCCECCCCCHHHHCCCCCCC

**Seq.** SSAELKAAFEAAKAKNWRFYKRDTVGNEEEV

**Proteus** cchhhhhhhhhhccccceeeccccccccccc

**SSpro** CCCCCCCCHHHHHHHCCEHEECCCCCCCCCC
